# Sparsentan as First-Line Treatment in IgAN From SPARTAN

**DOI:** 10.1016/j.ekir.2026.107105

**Published:** 2026-09-24

**Authors:** Chee Kay Cheung, William A. Barratt, Sulalita Chaki, Silpa Chinnakotla, Neeraj Dhaun, Matthew P.M. Graham-Brown, Siân Griffin, Bruce Hendry, Alexandra Howson, Amal A.A. Jama, Wenjun Ju, Ishika Khan, Radko Komers, Matthias Kretzler, Alex Mercer, Stephanie Moody, Viji Nair, Nadia Nawaz, Kelly Parke, Ian S.D. Roberts, Matthew Sayer, Smeeta Sinha, Justyna Szklarzewicz, Lisa Willcocks, Jonathan Barratt

**Affiliations:** 1 Department of Cardiovascular Sciences, University of Leicester, Leicester, UK; 2 John Walls Renal Unit, University Hospitals of Leicester NHS Trust, Leicester, UK; 3 Department of Internal Medicine, University of Michigan, Ann Arbor, Michigan, USA; 4 Institute for Neuroscience and Cardiovascular Research, Edinburgh Kidney, The Queen’s Medical Research Institute, University of Edinburgh, Edinburgh, Scotland, UK; 5 Department of Renal Medicine, Royal Infirmary of Edinburgh, Edinburgh, Scotland, UK; 6 Division of Cardiovascular Sciences, NIHR Leicester Biomedical Research Centre, Leicester, UK; 7 Department of Nephrology and Transplantation, University Hospital of Wales, Cardiff, Wales, UK; 8 Medical Affairs, Travere Therapeutics, Inc., San Diego, California, USA; 9 Renal Research Unit, University Hospitals of Leicester NHS Trust, Leicester, UK; 10 Clinical Development, Nephrology, Travere Therapeutics, Inc., San Diego, California, USA; 11 Medicine/Nephrology and Computational Medicine and Bioinformatics, University of Michigan, Ann Arbor, Michigan, USA; 12 Clinical Drug Development, JAMCO Pharma Consulting, Stockholm, Sweden; 13 Biostatistics, Travere Therapeutics, Inc., San Diego, California, USA; 14 Department of Internal Medicine, Division of Nephrology, University of Michigan, Ann Arbor, Michigan, USA; 15 Department of Cardiovascular Sciences, University Hospitals of Leicester NHS Trust, Leicester, UK; 16 Department of Cellular Pathology, Oxford University Hospitals NHS Foundation Trust, Oxford, UK; 17 Division of Cardiovascular Sciences, University of Manchester, UK; 18 Department of Renal Medicine, Donal O’Donoghue Renal Research Centre, Northern Care Alliance NHS Foundation Trust, Greater Manchester, UK; 19 Department of Renal Medicine, Cambridge University Hospitals NHS Trust, Cambridge, UK

**Keywords:** angiotensin II, endothelin-1, glomerulonephritis, IgA nephropathy, proteinuria, sparsentan

## Abstract

**Introduction:**

The PROTECT trial demonstrated that sparsentan reduces proteinuria and preserves kidney function in patients with IgA nephropathy (IgAN). However, its potential as first-line therapy requires further investigation. SPARTAN (NCT04663204) is an experimental study evaluating efficacy, safety, and mechanistic actions of sparsentan in incident patients with IgAN. We present findings from a prespecified 24-week interim analysis.

**Methods:**

A total of 12 adults with incident biopsy-proven IgAN, urine protein excretion (UPE) ≥ 0.5 g/d, estimated glomerular filtration rate (eGFR) ≥ 30 ml/min per 1.73 m^2^, and no renin-angiotensin system inhibitor (RASi) treatment within the previous 12 months were enrolled. Sparsentan was administered for 110 weeks with a 4-week safety follow-up. Assessments included proteinuria, eGFR, blood pressure (BP), body weight, total body water, cardiovascular measures, urinary biomarkers, and safety.

**Results:**

Mean age was 35.8 years, median (interquartile range [IQR]) UPE was 1.7 (0.6–3.3) g/d, and mean eGFR was 70.2 (SD: 25.0) ml/min per 1.73 m^2^ at baseline. Urine protein-to-creatinine ratio (UPCR) declined rapidly (−61.9% at week 4), and reductions were sustained over 24 weeks (−68.8%); 58% of patients achieved complete remission of proteinuria (<0.3 g/d) during treatment. After an initial decrease, BP remained stable with stable eGFR, body weight, and cardiovascular risk variables. Total body water decreased. Rapid and sustained reductions in urinary biomarkers of inflammation, immune cell recruitment and modulation, and fibrosis were observed. The most frequent adverse event was dizziness (50% patients); 1 patient discontinued because of hypotension.

**Conclusion:**

First-line sparsentan reduced proteinuria by approximately 70% over 24 weeks and was generally well tolerated. Biomarker observations support anti-inflammatory and antifibrotic effects of sparsentan beyond its hemodynamic effects.

## Graphical abstract

**Figure:**
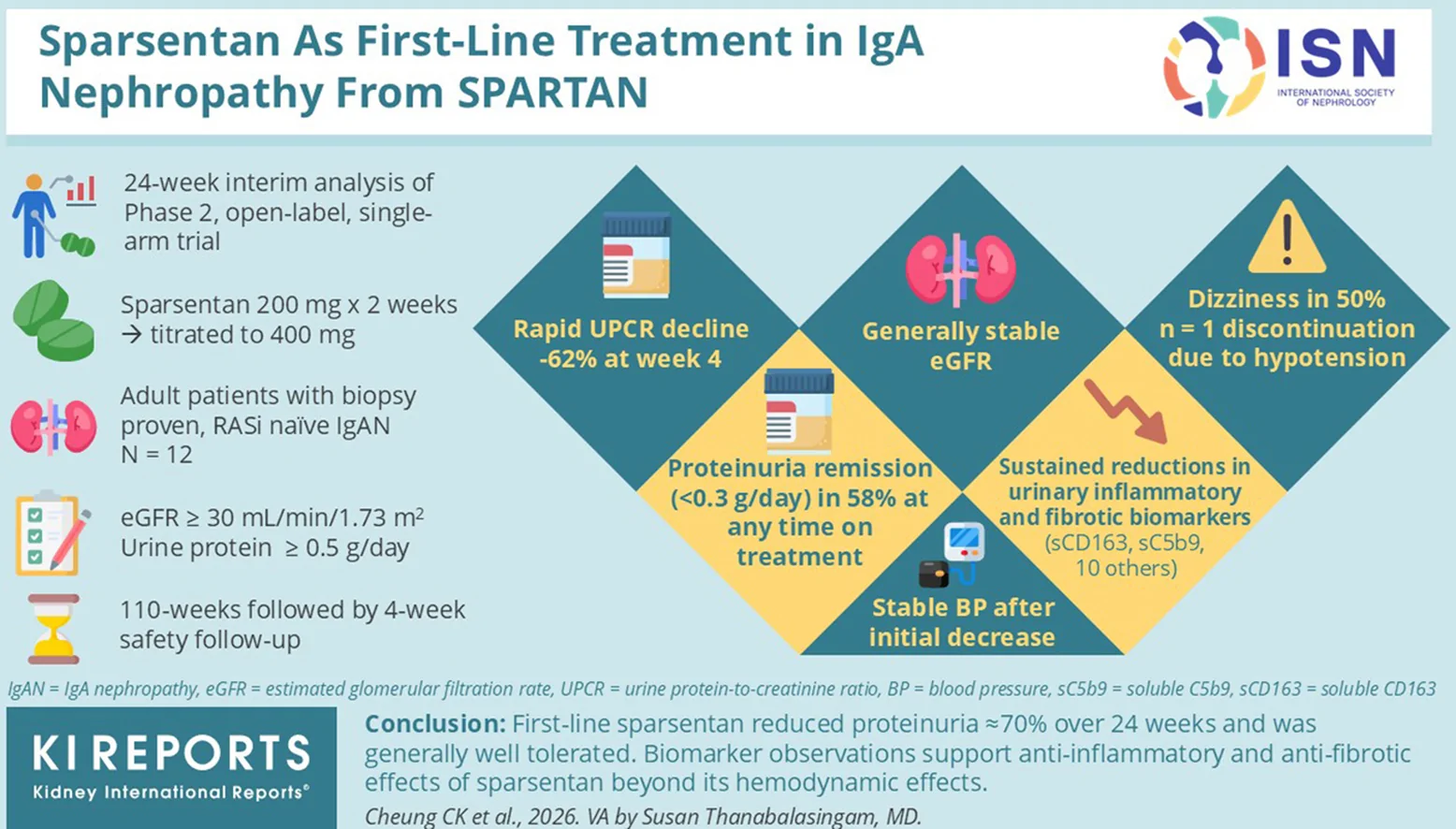

The treatment of IgAN is undergoing rapid change. Its poor long-term outcomes are now well recognized, with registry data from the UK National Registry of Rare Kidney Diseases study highlighting a median survival of 11.4 years from diagnosis to the combined end point of kidney failure or death in adults^1^ and other registries demonstrating similarly poor outcomes globally.^2^, ^3^, ^4^, ^5^, ^6^ Based on these data, the 2025 Kidney Disease: Improving Global Outcomes guideline highlighted a key treatment goal, to achieve an eGFR decline of < 1 ml/min per 1.73 m^2^ per year, in order to reduce the risk of patients reaching kidney failure within their expected lifetime.^7^ Because it is not possible to assess this goal in a clinic visit-to-visit basis, targeting a reduction of proteinuria, the only validated early biomarker for disease progression, to <0.5 g/d or ideally <0.3 g/d (complete remission), was highlighted as an appropriate treatment goal in IgAN.^7^

The therapeutic armamentarium is rapidly expanding, with 6 novel treatments recently approved specifically for IgAN.^8^, ^9^, ^10^, ^11^, ^12^, ^13^, ^14^, ^15^, ^16^ Furthermore, there are several other agents in late-phase clinical development that target IgA-producing B cells and plasma cells, complement activity, and endothelin signaling.^7^^,^^17^^,^^18^ To use these therapies effectively, a better understanding of how they work and of biomarkers that can help predict and assess treatment responses is required to individualize and optimize treatment.^19^^,^^20^

To date, all Phase 3 randomized controlled trials in IgAN have enrolled prevalent patients who have elevated levels of proteinuria despite receiving optimized RASi as first-line therapy, in accordance with the previous standard of care.^21^ However, recent observational data suggest that the sooner low levels of proteinuria are achieved, the lower the risk of disease progression.^22^^,^^23^ These observations raise the question of whether emerging therapies for IgAN could serve as first-line treatment options to rapidly lower proteinuria and provide better outcomes than the established standard of care.

The Phase 3 PROTECT trial demonstrated that treatment with sparsentan, a non-immunosuppressive, dual endothelin angiotensin receptor antagonist, was superior to irbesartan in lowering proteinuria and slowing kidney function decline over a 2-year period, and was well tolerated.^21^^,^^24^ The trial enrolled patients with a median duration of 4 years since diagnosis.^21^ In the SPARTAN trial (ClinicalTrials.gov, NCT04663204), we investigated the effectiveness and safety of sparsentan as a first-line therapy in incident IgAN patients (<6 months from biopsy) without previous RASi treatment. Mechanistic aspects of sparsentan treatment were examined to determine how sparsentan targets the underlying pathophysiology of IgAN. Here we report findings from an interim analysis over the first 24 weeks of treatment.

## Methods

### Study Design and Eligibility Criteria

SPARTAN (NCT04663204) is a Phase 2, open-label, single-arm, multicenter, experimental study being conducted at 5 hospitals in the United Kingdom. After screening, the study comprises a treatment period with sparsentan for 110 weeks with a 4-week follow-up period without sparsentan during which patients may start standard-of-care treatment (114 weeks total) (Figure 1).

**Figure 1:**
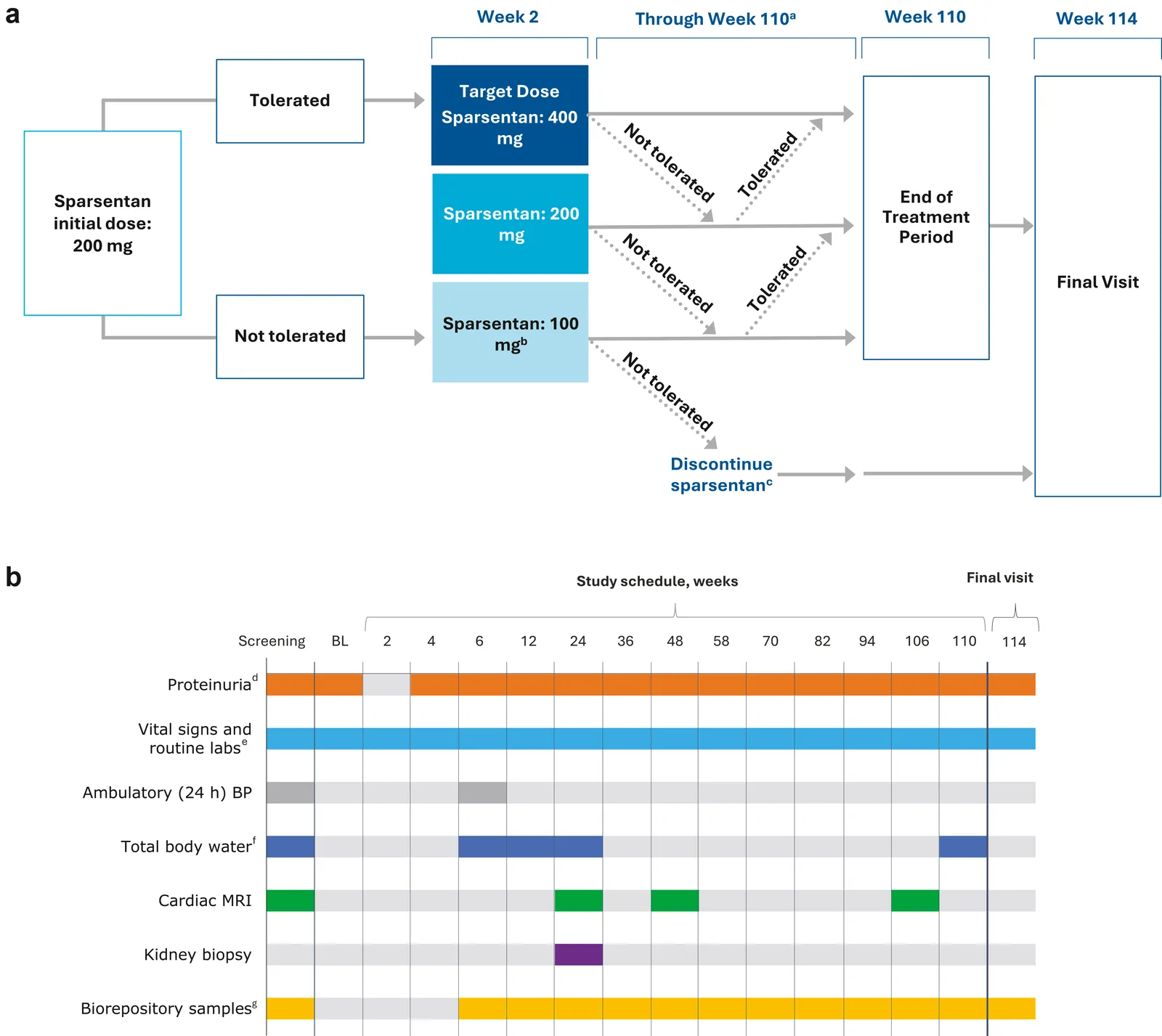
Study design and assessment schedule. (a) Study design and dosing titration. (b) Schedule of trial assessments. BP, blood pressure; MRI, magnetic resonance imaging. ^a^Reductions from the target dose back to the initial dose (or from the initial dose to 100 mg) are permitted at any time after titrating up to the target dose; similarly, titrations up to the target dose are permitted at subsequent visits. ^b^Patients who do not tolerate the initial dose for any reason may have their dose reduced to 100 mg at any time prior to or at the week 2 visit. ^c^Patients who do not tolerate the 100-mg dose for any reason may discontinue sparsentan and will be placed on appropriate standard-of-care treatment. ^d^With 24-hour collection. ^e^Vital signs include office BP and weight, and routine laboratory studies include eGFR, total cholesterol, HDL cholesterol, non-HDL cholesterol, LDL cholesterol, triglycerides, blood glucose, and NT-proBNP. ^f^Measured by bioimpedance spectroscopy. ^g^Urine, plasma, and serum.

This study was conducted in compliance with all applicable laws and statutes and all relevant guidance and principles of International Council for Harmonization, Good Clinical Practice guidelines, and the Declaration of Helsinki. The protocol, informed consent form, and any information provided to patients were approved by the appropriate research ethics committee, the health research authority, and applicable regulatory authorities. All patients enrolled provided signed written informed consent. Patients included in the study were adults (≥18 years old) with biopsy-proven IgAN (diagnosed within 6 months of enrollment) who had not received angiotensin-converting enzyme inhibitors or angiotensin receptor blockers therapy in the previous 12 months and had not received any systemic immunosuppressive therapy within the previous 6 months. Other key inclusion criteria were a UPE of ≥0.5 g/d, an eGFR of ≥30 ml/min per 1.73 m^2^, a systolic BP of 110 to 150 mm Hg, and a diastolic BP of 60 to 100 mm Hg.

Patients were excluded from the study if they had IgAN secondary to another condition or rapidly progressive glomerulonephritis. Full details on inclusion and exclusion criteria are listed in the Supplementary Methods.

After enrollment, patients received sparsentan 200 mg/d for the first 2 weeks. At the end of week 2, the dose was titrated to the target of 400 mg/d. Patients unable to tolerate the target dose could have their dose reduced at any time; similarly, titrations up to the target dose were permitted at subsequent visits (Figure 1a). Further details on the dose tolerability and titration are outlined in the Supplementary Methods.

### Assessments

Study visits occurred at screening, baseline (day 1), and designated weeks as outlined in Figure 1b. This interim analysis covered study visits up to week 24 except for eGFR, which is presented up to week 36. A medical history, including 12-lead electrocardiogram, was performed at screening. The sex of each patient was obtained from the patient at screening. The schedule of routine trial assessments including physical examinations, vital signs, and routine blood and urine samples is summarized in Figure 1b. Proteinuria was determined from 24-hour urine sample collection. eGFR for each visit was determined using the 2009 Chronic Kidney Disease Epidemiology Collaboration formula.^25^ BP was measured after patients had been sitting comfortably in a chair for at least 5 minutes before obtaining 3 readings. Ambulatory BP was performed over a 24-hour period at specified visits. Total body water was assessed via bioelectrical impedance spectroscopy (bioimpedance).

Urinary biomarkers from biorepository samples were measured by enzyme-linked immunosorbent assay with urine data normalized to creatinine; details are provided in the Supplementary Methods. The methodology used for the protein-protein network mapping is summarized in the Supplementary Methods.

Cardiac magnetic resonance imaging (MRI) examined right and left ventricular structure and function, aortic distensibility and pulse wave velocity, native T1 and T2 mapping (to assess diffuse myocardial fibrosis and edema, respectively),^26^ and longitudinal circumferential strain. For those patients with an eGFR > 60 ml/min per 1.73 m^2^, late gadolinium-enhanced imaging, post contrast T1 mapping, and extracellular volume MRI were performed. MRI was performed at participating research sites or at University Hospitals of Leicester National Health Service Trust. Details for acquisition and analysis of cardiac and renal MRI scans are included in the Supplementary Methods.

Whole slide images of diagnostic kidney biopsy slides were scored by a single pathologist for MEST-C (M = mesangial hypercellularity, E = endocapillary proliferation, S = segmental glomerulosclerosis, T = tubular atrophy/interstitial fibrosis, and C = crescents) according to the Oxford Classification.^27^

### Clinical End Points

The primary objective was to evaluate the safety and tolerability of sparsentan in incident patients with IgAN. Treatment responses to sparsentan in this interim analysis were assessed by changes from baseline in proteinuria (UPCR and UPE), the proportion of patients who achieved complete remission of proteinuria (UPE < 0.3 g/d) at least once during the treatment period, and mean eGFR. Key safety end points included the proportion of patients with abnormalities in clinical laboratory assessments, vital signs, and occurrence of adverse events.

Exploratory end points included change from baseline in 24-hour ambulatory BP at week 6 as well as changes in total body water (measured by bioimpedance spectroscopy), biomarkers in plasma, serum, and urine, and cardiac function (measured by MRI).

### Statistical Analysis

The number of patients to be enrolled into this trial was based on clinical feasibility and what was judged by the investigators to be sufficient to provide initial insights into the safety and efficacy of sparsentan in incident patients with IgAN; no formal power calculations were performed. Demographics and baseline characteristics are reported for the full analysis set (i.e., all patients who were enrolled and received ≥ 1 dose of sparsentan).

Proteinuria (UPE and UPCR) and cardio-renal status and risk parameters (eGFR, BP, body weight, total body water, N-terminal pro-B-type natriuretic peptide, lipids, and blood glucose) are reported using the full analysis set using on-treatment data. One patient discontinued at week 6 and was not included as part of these analyses after week 6. Data for exploratory end points including 24-hour ambulatory BP, urinary biomarkers, and observations from cardiac MRI are reported using on-treatment data for only patients who completed all 24 weeks of treatment.

Descriptive statistics of observed value and percent change from baseline (including geometric mean) by visit is presented for UPE and UPCR. The number and proportion of patients achieving UPE of < 0.3 g/d (complete remission of proteinuria) is presented descriptively by visit. Descriptive summary statistics and change from baseline are presented for the other primary and exploratory end points included in this study (Clinical End Points section). For all analyses, nominal visit is used to summarize by visit values.

MRI summaries were obtained using SPSS (version 29.0; IBM, Armonk, NY).

Safety was analyzed using descriptive summary statistics. Treatment-emergent adverse events were coded using the Medical Dictionary for Regulatory Activities (version 23.0).

All statistical analyses, except where specified above for MRI, were performed in accordance with the International Council for Harmonisation of Technical Requirements for Pharmaceuticals for Human Use E9: Statistical Principles for Clinical Trials, using SAS (version 9.4 or higher; SAS Institute Inc., Cary, NC) or equivalent statistical reporting software.

## Results

### Patient Characteristics

As of the data cutoff for this interim analysis of February 15, 2024, 12 patients were enrolled and received treatment with sparsentan; most were male (58%) and White (83%). Median (IQR) time from initial kidney biopsy to informed content was 13 (7–20) weeks. The mean (SD) age was 35.8 (12.2) years and mean (SD) eGFR at baseline was 70.2 (25.0) ml/min per 1.73 m^2^. Median (IQR) UPE and UPCR at baseline were 1.7 (0.6–3.3) g/d and 1.3 (0.4–1.7) g/g, respectively. Demographic, clinical, histological, and biochemical characteristics at baseline are shown in Table 1. The median (IQR) duration of treatment at the interim efficacy analysis cutoff was 24 (23.5–25.5) weeks. All patients received at least 1 dose of sparsentan, and 9 patients were titrated to the target dose of 400 mg. Of note, 3 patients remained on 200 mg of sparsentan, likely because of tolerability considerations related to low BP. One of these patients subsequently had their sparsentan dose down titrated from 200 to 100 mg; however, this patient discontinued early because of hypotension after week 6 but remained on study. Eleven patients (91.7%) continued to receive sparsentan (Supplementary Figure S1).

**Table 1:** Baseline characteristics (full analysis set)

Characteristic	Sparsentan (*N* = 12)
Age at informed consent, mean (SD), yr	35.8 (12.2)
Sex, *n* (%)	
Male	7 (58)
Female	5 (42)
Race, *n* (%)	
Asian	0
Black	0
White	10 (83)
Other	2 (17)
Time from initial kidney biopsy to informed consent, median (IQR), weeks	13 (7–20)
Urine albumin-to-creatinine ratio, median (IQR), g/g	0.6 (0.2–0.8)
Urine protein-to-creatinine ratio, median (IQR), g/g	1.3 (0.4–1.7)
Urine protein excretion, median (IQR), g/d	1.7 (0.6-3.3)
Serum albumin, mean (SD), g/l	37.8 (5.3)
Hematuria, *n* (%)^a^	
Absent	0
Trace	0
+1	2 (17)
+2	0
+3	10 (83)
eGFR, mean (SD), ml/min per 1.73 m^2^	70.2 (25.0)
Seated office BP, mean (SD), mm Hg	
Systolic	125 (10)
Diastolic	78 (10)
Total body water, mean (SD), l	47.1 (7.4)
Weight, mean (SD), kg	83.1 (24.7)
BMI, mean (SD), kg/m^2^	27.5 (7.2)
NT-proBNP, mean (SD), ng/l	135.3 (166.9)
Lipids, mean (SD), mmol/l	
Total cholesterol	4.4 (0.7)
HDL cholesterol	1.3 (0.4)
Non-HDL cholesterol	3.5 (0.8)
LDL cholesterol	2.8 (0.7)
Triglycerides	1.5 (0.7)
Blood glucose, mean (SD), mmol/l^b^	4.7 (0.6)
Urinary biomarkers (*n* = 11), mean (SD)^c^	
BAFF	154 (115.8) pg/mg^d^
sC5b9	13449 (12422.9) pg/mg^d^
sCD163	3943 (4055.4) pg/mg^d^
α2M	3000 (3733.2) ng/mg^e^
CHI3L1	1003 (837.9) pg/mg^d^
Clusterin	527 (439.0) ng/mg^e^
GDF15	12688 (13599.4) pg/mg^d^
Plasminogen	335 (397.9) ng/mg^e^
CXCL10	156 (106.6) pg/mg^d^
CXCL16	5983 (7105.2) pg/mg^d^
IL6	65 (78.9) pg/mg^d^
MCP-1	814 (783.4) pg/mg^d^
Baseline concomitant medication use, *n* (%)	
Antihypertensive medications	0
Lipid-lowering medications	1 (8.3)
Blood glucose control medications	0
Comorbidities of interest, *n* (%)	
Hypertension	0
Diabetes	0
Oxford classification (MEST-C), *n* (%)	
Mesangial hypercellularity (M1)	3 (25)
Endocapillary hypercellularity (E1)	2 (17)
Segmental glomerulosclerosis (S1)^f^	10 (83)
Tubular atrophy/interstitial fibrosis (T1, T2)	3 (25), 1 (8)
Cellular or fibrocellular crescents (C1)	1 (8)

α2M, alpha-2-macroglobulin; BAFF, B-cell activating factor; BMI, body mass index; BP, blood pressure; CHI3L1, chitinase-3-like protein 1; CXCL10, CXC motif chemokine ligand 10; CXCL16, CXC motif chemokine ligand 16; eGFR, estimated glomerular filtration rate; GDF15, growth/differentiation factor 15; HDL, high-density lipoprotein; IL6, interleukin-6; LDL, low-density lipoprotein; MCP-1, monocyte chemoattractant protein-1; MEST-C, M = mesangial hypercellularity, E = endocapillary proliferation, S = segmental glomerulosclerosis, T = tubular atrophy/interstitial fibrosis, C = crescents; NT-proBNP, N-terminal pro-B-type natriuretic peptide; sC5b9, soluble C5b9; sCD163, soluble CD163.

a Hematuria measured by dipstick test.

b Based on random nonfasting blood glucose measurements.

c One patient discontinued after week 6 and was excluded from the analysis; *n* = 11.

d pg of biomarker per mg of creatinine.

e ng of biomarker per mg of creatinine.

f One patient had an S1 lesion with podocytopathic features.

### Efficacy and Exploratory Outcomes

#### Proteinuria

A rapid and sustained reduction in proteinuria was achieved with sparsentan treatment, with a geometric mean percent change in UPCR from baseline of −61.9% by week 4 and −68.8% by week 24 (Figure 2). Mean (SD) UPCR decreased from 1.4 (1.1) g/g at baseline to 0.4 (0.3) g/g at week 24 (Supplementary Figure S2). Complete remission of proteinuria at any time over the 24-week treatment period occurred in 7 (58%) patients. Of note, 5 patients had UPE of > 2 g/d at baseline. Of those 5 patients, 4 had proteinuria reductions of ≥ 75% at any time during the first 24 weeks of treatment (Supplementary Figure S3).

**Figure 2:**
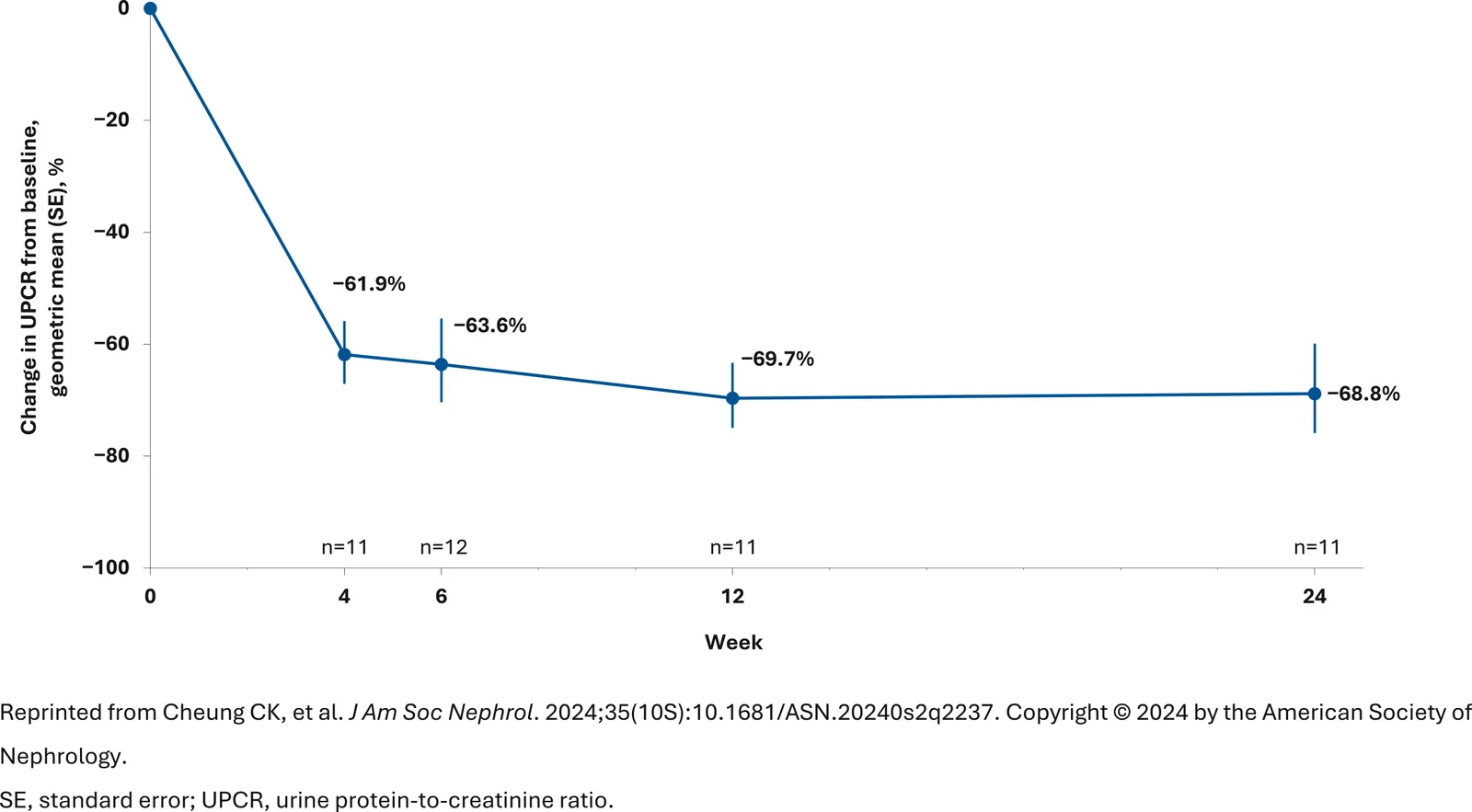
Geometric mean (SE) percent change from baseline in UPCR at each study visit. Reprinted from Cheung CK, et al. *J Am Soc Nephrol.* 2024.^28^ Copyright © 2024 by the American Society of Nephrology.

#### eGFR

eGFR values at each visit remained relatively stable. There was no evidence for a marked acute lowering effect on eGFR over the initial weeks of treatment (Supplementary Figure S4).

#### Hematuria

All patients had hematuria at baseline (*N* = 12; trace, *n* = 0 [0%]; 1+, *n* = 2 [17%]; 2+, *n* = 0 [0%]; 3+, *n* = 10 [83%]) and at week 24 (*N* = 11; trace, *n* = 0 [0%]; 1+, *n* = 1 [9%]; 2+, *n* = 2 [18%]; 3+, *n* = 8 [73%]).

#### Urinary Biomarker Observations

Rapid and sustained reductions were observed in several urinary biomarkers after initiation of sparsentan treatment (Figure 3). B-cell activating factor and soluble C5b9, associated with immune cell modulation and complement activation, were reduced by 76% (standard error of the mean, 5) and 68% (standard error of the mean, 18) from baseline, respectively (Figure 3a and b). Inflammatory and profibrotic biomarkers including soluble CD163 (a marker of alternatively activated macrophages), alpha-2-macroglobulin, chitinase-3-like protein 1 (also known as YKL-40), clusterin, growth differentiation factor 15, and plasminogen were reduced ranging from 42% to 91% (Figure 3c–h). Lesser reductions (15%–30%) in other chemokine and cytokine biomarkers involved in immune cell recruitment (C-X-C motif chemokine ligand 10, C-X-C motif chemokine ligand 16, interleukin-6, monocyte chemoattractant protein-1) were also observed after starting sparsentan treatment (Figure 3i–l). Absolute values for urinary biomarkers are presented in Supplementary Figure S5.

**Figure 3:**
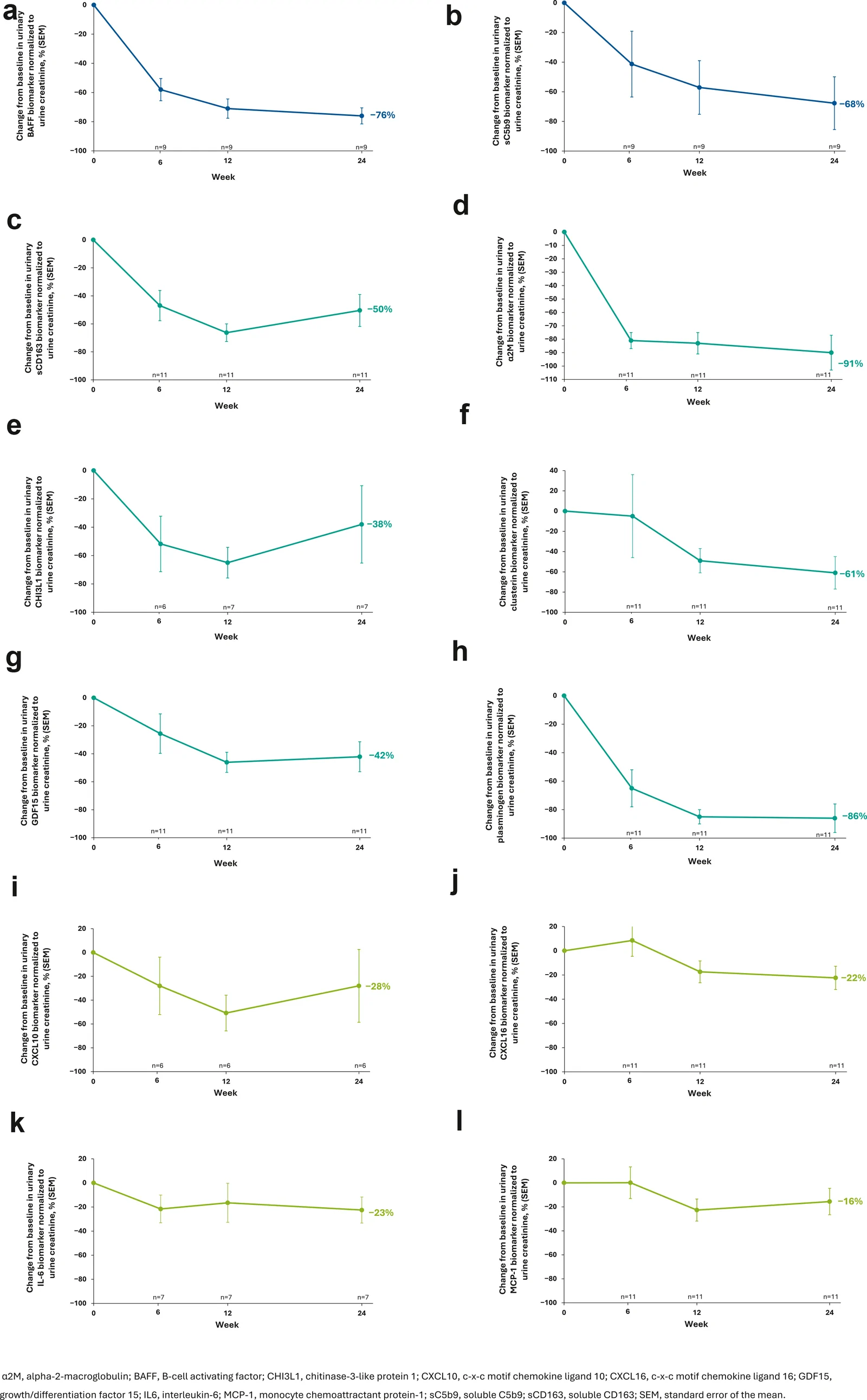
Change from baseline in urine biomarkers at each available study visit. Mean (SEM) percentage change in (a) BAFF, (b) sC5b9, (c) sCD163, (d) α2M, (e) CHI3L1, (f) clusterin, (g) GDF15, (h) plasminogen, (i) CXCL10, (j) CXCL16, (k) IL6, and (l) MCP-1 at each study visit. Blue, immune cell modulation biomarkers; teal, inflammatory and profibrotic biomarkers; green, chemokine and cytokine biomarkers.

Protein-protein network mapping revealed a close relationship between affected biomarkers, suggesting a coordinated effect of sparsentan on modulation of intrarenal inflammatory and fibrotic pathways within multiple nephron segments (Supplementary Figure S6).

#### Cardiovascular Risk Variables

After an initial decrease, office BP remained stable during the rest of the treatment period (Figure 4a). Mean baseline systolic and diastolic BPs were 125 and 78 mm Hg, with a mean (SD) change from baseline of −15 (6) mm Hg and −7 (9) mm Hg at week 24, respectively. A comparison of office and 24-hour ambulatory BP showed similar reductions from baseline for both systolic and diastolic BP at week 6 (Supplementary Figure S7).

**Figure 4:**
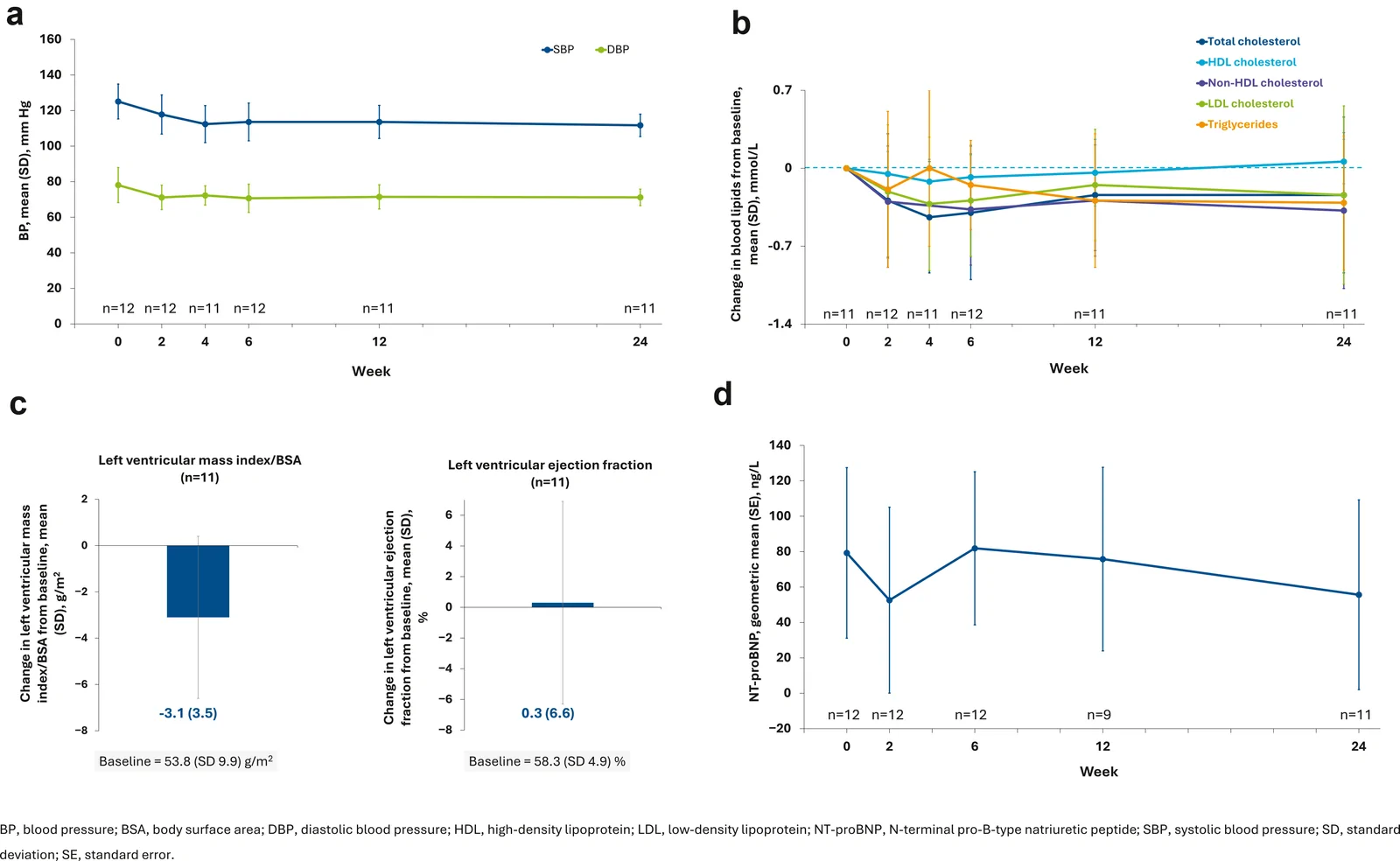
Mean change in cardiac-related variables. (a) Mean (SD) office BP at each study visit. (b) Mean (SD) change from baseline in lipid variables at each study visit. (c) Mean (SD) change from baseline in left ventricular mass and left ventricular ejection fraction at week 24. (d) Geometric mean (SE) change from baseline in NT-proBNP at selected study visits.

Measured blood lipid variables showed a trend toward improvement. The mean (SD) change from baseline to week 24 for total cholesterol, non-high-density lipoprotein cholesterol, low-density lipoprotein, and triglycerides were −0.24 (0.7), −0.38 (0.7), −0.24 (0.8), and −0.31 (0.6) mmol/l, respectively. The mean (SD) change from baseline in high-density lipoprotein was 0.06 (0.2) mmol/l (Figure 4b). Changes in blood glucose levels were minimal, with a mean (SD) change from baseline to week 24 of 0.2 (0.6) mmol/l. Mean blood glucose at baseline and week 24 were 4.7 (0.6) and 4.8 (0.3) mmol/l, respectively (Supplementary Table S1).

Compared with baseline there were no statistically significant changes in the cardiac structural (mass, T1, T2, or volume) or functional (ejection fraction or strain) measures in the MRI scans (Supplementary Table S2). A reduction in mean (SD) left ventricular mass (indexed to body surface area) (−3.1 [3.5] g/m^2^) and minimal change in mean (SD) left ventricular ejection fraction (0.3% [6.6%]) were observed at week 24, but these changes were not statistically significant (Figure 4c). Mean (SD) changes in native T1 (12.9 [35.1] ms) and native T2 (2.3 [3.8] ms) were also not statistically significant (Supplementary Table S2).

N-terminal pro-B-type natriuretic peptide levels initially declined at week 2 and remained relatively stable throughout the treatment period (Figure 4d). Body weight remained stable, with the mean (SD) change from baseline to week 24 being –1.0 (3.3) kg (Supplementary Table S1). Mean (SD) total body water at baseline was 47.1 (7.4) l, and modest reductions were observed at weeks 6 (−2.0 [7.2] l) and 24 (−2.4 [7.4] l) (Supplementary Table S1).

#### Safety

Sparsentan was generally well tolerated during the treatment period, with no new safety signals identified (Table 2). One patient permanently discontinued treatment because of hypotension after week 6. The only serious adverse event reported was an infected foot abscess, in a patient who had a chronic foot ulcer predating entry into the study. Acute kidney injury occurred in the same patient secondary to the foot ulcer infection. There were 2 cases of hyperkalemia; 1 was mild and considered unlikely related to treatment, whereas the other was moderate in severity and considered possibly related to sparsentan. Both resolved without modification of sparsentan dosing or the need for initiation of potassium binders. There were no episodes of edema and no abnormal liver function tests.

**Table 2:** Any AEs, SAEs, and most common AEs (full analysis set)

AE parameter	Sparsentan (*N* = 12) *n* (%)
Any AE	12 (100)
Any SAE	1 (8)
Most common AEs (> 2 patients)	
Dizziness	6 (50)
Urinary tract infection	3 (25)
Dyspepsia	3 (25)
Vomiting	3 (25)

AE, adverse event; SAE, serious adverse event.

## Discussion

This interim analysis of the SPARTAN study demonstrated that first-line treatment with sparsentan in newly diagnosed patients with IgAN was associated with rapid and sustained reductions in proteinuria and was well tolerated. Kidney function remained stable over the follow-up period. Long-term eGFR data from the full 2-year study will be reported later. Reduction in biomarkers associated with kidney inflammation, immune cell activation, complement activity, and fibrosis in IgAN was also observed following sparsentan initiation. These findings highlight the potential beneficial intrarenal effects of dual endothelin-angiotensin blockade that extend beyond its known favorable hemodynamic effects.

Notably, the reduction in UPCR observed with sparsentan in the SPARTAN study (−68.8% at 24 weeks) was greater than that reported in the PROTECT trial (−49.8% at 36 weeks).^24^ This could be due in part to patients enrolled in PROTECT having received previous treatment with RASi and therefore having some degree of proteinuria reduction as well as other factors related to patient selection.^21^^,^^24^ Although early initiation of sparsentan could plausibly provide more rapid and profound proteinuria control in incident patients with IgAN, this hypothesis warrants further investigation. Participants in SPARTAN were younger and, on average, had somewhat more preserved kidney function and lower BP than those in PROTECT.^21^^,^^29^ These differences should be considered in the context of the incident SPARTAN population, in whom the median time from diagnosis to study entry was 3 months compared with approximately 4 years in PROTECT.^21^ Nevertheless, SPARTAN encompassed a range of disease severity and, despite differences between the study populations, was broadly comparable to PROTECT with respect to several important baseline disease characteristics.^29^ In particular, median baseline UPE levels were similar in SPARTAN and PROTECT (1.7 [IQR, 0.6–3.3] g/d vs. 1.8 [IQR, 1.3–2.8] g/d, respectively),^29^ as were diagnostic biopsy MEST-C scores (SPARTAN M1 [25%], E1 [17%], S1 [83%], T1 [25%], T2 [8%], C1 [8%] vs. PROTECT [*n* = 89] M1 [12%], E1 [22%], S1 [80%], T1 [23%], T2 [4%], C1 [21%]).^30^ Furthermore, findings from National Registry of Rare Kidney Diseases demonstrated that patients with IgAN and proteinuria of 0.5 to < 1.0 g/d remained at substantial risk of kidney failure,^1^ indicating that patients in SPARTAN with proteinuria at these levels were at risk of disease progression.

Strikingly, within 24 weeks of initiating sparsentan, nearly 60% (7 of 12) of patients in SPARTAN achieved complete remission of proteinuria (UPE < 0.3 g/d), a treatment goal recommended within the 2025 Kidney Disease: Improving Global Outcomes guideline.^7^ The clinical importance of achieving complete remission of proteinuria is underscored by analyses from PROTECT, which showed that attainment of this proteinuria target was associated with a lower incidence of the composite kidney failure end point (defined as 40% reduction in eGFR, end-stage kidney disease, or death).^31^

The urinary biomarker data from SPARTAN is particularly intriguing and warrants further study. Levels of urinary sCD163, a marker for alternatively activated macrophages that has been correlated with kidney macrophage infiltration and active lesions in IgAN,^32^ was reduced by approximately 50% by week 24. In the TESTING study, methylprednisolone reduced urinary sCD163 by 79% at 6 months in patients with IgAN, and it was shown that a ≥ 50% reduction in urinary sCD163 from baseline was associated with better kidney survival.^32^ Reductions in other inflammatory and profibrotic mediators were also observed in SPARTAN. Our findings are consistent with preclinical work that highlighted that sparsentan reduced glomerular macrophage infiltration, immune complex–mediated mesangial hypercellularity, and attenuated the expression of inflammatory genes.^33^, ^34^, ^35^ In the gddY spontaneous mouse model of IgAN, sparsentan treatment resulted in greater reductions in proteinuria and improved preservation of podocyte number and glycocalyx versus losartan, despite equivalent reductions in BP.^34^ The endothelin type A receptor is expressed by several immune cells, including macrophages,^36^ and therefore our findings are consistent with sparsentan having renal benefit via multiple pleiotropic effects known to be a result of dual angiotensin II and endothelin-1 inhibition. Our initial findings will be explored further in the repeat kidney biopsies taken in this study, which will examine transcriptomics by bulk single nuclear RNA sequencing and digital spatial profiling, as well as proteomic profiling of serum and urine samples.

The SPARTAN study is too small to draw definitive conclusions about the effects of sparsentan on cardiovascular risk parameters. The overall cardiovascular consequences of sparsentan treatment are likely to be driven by improvements in proteinuria and BP, along with preservation of eGFR over time; each of these outcomes supports cardiovascular benefit. It is also reassuring that key blood lipid indices of cardiovascular risk were either stable or trended favorably on initiation of sparsentan treatment. Selective endothelin receptor antagonism has been reported to reduce circulating proprotein convertase subtilisin/kexin type 9 levels and improve lipid parameters in a small study with patients with chronic kidney disease, suggesting a potential interaction between endothelin signaling and lipid regulatory pathways.^37^

Consistent with previous studies in patients with IgAN,^21^^,^^24^ sparsentan was generally well tolerated in the SPARTAN trial. Although hypotension was reported to be the most frequent adverse event, most cases were manageable with dose reduction, and only 1 patient discontinued treatment because of hypotension. Traditionally, endothelin receptor antagonism has been associated with fluid retention.^38^^,^^39^ However, with sparsentan, a dual endothelin angiotensin receptor antagonist, there was no indication of fluid retention based on changes in body weight, bioimpedance measurements, and N-terminal pro-B-type natriuretic peptide in this interim analysis. These findings align with observations from other Phase 2 and 3 studies, showing a low risk of fluid retention with sparsentan in patients with glomerular disease.^21^^,^^24^^,^^40^, ^41^, ^42^ Although fluid retention is a recognized risk with single-target endothelin antagonists, the minimal changes in fluid status reported with the dual endothelin angiotensin receptor antagonist sparsentan may be due to specific pharmacokinetic characteristics of sparsentan as a single molecule with different affinities towards angiotensin II type 1 and endothelin type A receptors.^35^^,^^43^

There are certain limitations to this study. First, a small number of study participants were included, the enrolled population was primarily White, and the follow-up period reported was relatively short. We will report the full 2-year findings, including eGFR data, upon completion of the study. Additionally, enrollment in SPARTAN took place before the widespread use of sodium-glucose cotransporter-2 inhibitors (SGLT2is) for IgAN. No participants were receiving SGLT2i at baseline or initiated therapy within the first 6 months of the trial; as such, the results of this interim analysis are not confounded by SGLT2i use, although 3 patients initiated SGLT2i later during follow-up, reflecting this change in clinical practice. Similarly, there was no initiation of corticosteroids or other immunosuppressive therapy.

With several Phase 3 trials of novel therapies in IgAN reporting beneficial effects in terms of proteinuria reduction and slowing kidney function decline,^7^ there is growing interest in understanding how these novel therapies work, and when and in whom they can be best used.^44^ The SPARTAN trial was designed to act as a discovery study to identify candidate biomarkers that can be subsequently validated in biorepositories from larger Phase 3 studies and compared with other kidney disease cohorts. However, in the absence of a control group, changes in biomarkers observed after sparsentan initiation should be considered exploratory. To this end, we are currently examining the effects of sparsentan treatment on the biomarkers identified in this study in the PROTECT (IgAN) and DUPLEX (focal segmental glomerulosclerosis) cohorts to validate our findings in larger cohorts against active control groups and determine if the effects are specific to IgAN or more broadly applicable to other forms of kidney disease. This approach should shed further light both on the actions of sparsentan and the underlying disease pathogenesis. Mechanistic studies that include repeat kidney biopsies and biomarker discovery are now in progress for other therapies for IgAN.^45^, ^46^, ^47^, ^48^, ^49^

The 2025 Kidney Disease: Improving Global Outcomes IgAN guideline states that the observed greater reduction in proteinuria and eGFR preservation with combined dual blockade of the renin-angiotensin and endothelin systems are associated with improved outcomes and suggest that this approach may be a more effective first-line treatment to manage the responses to IgAN-induced nephron loss than RASi.^7^ The interim findings from SPARTAN in relation to sparsentan’s effects on proteinuria, eGFR, and potential anti-inflammatory and antifibrotic effects add further evidence in support of this approach.

## Disclosure

CKC has received consulting fees from Alexion Pharmaceuticals, Boehringer Ingelheim, Calliditas Therapeutics, CSL Vifor, Dimerix Limited, Emerald Clinical, Novartis, Otsuka Pharmaceuticals, Roche, Stada Arzneimittel AG, Travere Therapeutics, Inc., and Vera Therapeutics; and research funding from Travere Therapeutics, Inc. ND has received consulting fees from Travere Therapeutics, Inc., research funding from Pfizer and Travere Therapeutics, Inc., and serves as an advisor for Travere Therapeutics, Inc. MGB has received research funding from Kidney Research UK, British Heart Foundation, and Stoneygate Trust and has received honoraria from Astellas, Boehringer Ingelheim, AstraZeneca, Novartis, and OmniaMed. SG has received consulting fees from Alexion Pharmaceuticals, Bayer, CSL Vifor, Eledon Pharmaceuticals, Inc., Hansa Biopharma, and Takeda Pharmaceuticals; honoraria from Bayer, CSL Vifor, and Travere Therapeutics, Inc.; and serves as part of speakers’ bureaus for CSL Vifor and Travere Therapeutics, Inc. BH is an employee and shareholder of Travere Therapeutics, Inc. WJ has received consulting fees from Travere Therapeutics, Inc. WJ and MK hold a patent PCT/EP2014/073413 “Biomarkers and methods for progression prediction for chronic kidney disease.” RK is an employee and shareholder of Travere Therapeutics, Inc. MK has received consulting fees from the Alport Foundation, Boehringer Ingelheim, Certa, Chan Zuckerberg Initiative, Chinook Therapeutics, Dimerix Limited, European Union Innovative Medicine Initiative, Maze Therapeutics, National Institutes of Health, Novo Nordisk, Renalytix AI, Regeneron Pharmaceuticals, Roche (Genentech), Sanofi, Travere Therapeutics, Inc., and Vera Therapeutics. AM is an employee of JAMCO Pharma Consulting AB; reports consulting fees from Advanz Pharma, Biogen, CSL Vifor; Dimerix, Timberlyne Therapeutics, Travere Therapeutics, Inc., and Vera Therapeutics; and has ownership interest in JAMCO Pharma Consulting AB. SM is an employee and shareholder of Travere Therapeutics, Inc. IR serves as Director of UK Renal Pathology Limited; reports consultancy agreements with Alexion, Astellas, CSL Vifor, Novartis, Otsuka, Purespring Therapeutics, Sobi, Travere Therapeutics, Inc., and Vera Therapeutics; and serves as Scheme Organiser for UK National Renal Pathology EQA Scheme. MS has received research funding from Travere Therapeutics, Inc. SS has received consulting fees from Inozyme Pharma and Sanifit Therapeutics S.A.; research funding from AstraZeneca, CSL Vifor, Johnson & Johnson, and Sanofi Genzyme; honoraria from AstraZeneca, Bayer, Boehringer Ingelheim, CSL Vifor, GSK, Medscape, Menarini Group, Napp Pharmaceuticals, Novartis, and Sanofi Genzyme; is the National Clinical Director of Renal Medicine for NHS England; and is a trustee of the Kidneys for Life Charity. JS has received consultancy fees from Timberlyne Therapeutics and Travere Therapeutics, Inc. and has been part of advisory boards for CSL Vifor. LW has received consulting fees from CSL Vifor, Novartis, and Travere Therapeutics, Inc.; research funding from the Biomedical Research Council and the National Institute for Health and Care Research; honoraria from Otsuka Pharmaceuticals; has been part of advisory boards for CSL Vifor, Eledon Pharmaceuticals, Inc., and Sobi; and serves as part of speakers bureaus for CSL Vifor, Novartis, and Otsuka Pharmaceuticals. JB has received consulting fees from Alnylam Pharmaceuticals, argenx, Astellas Pharma, Inc., BioCryst Pharmaceuticals, Calliditas Therapeutics, Chinook Therapeutics, Dimerix Limited, Galapagos, Novartis, Omeros, Otsuka (Visterra) Pharmaceuticals, Travere Therapeutics, Inc., and Vera Therapeutics; and research funding from argenx, Calliditas Therapeutics, Chinook Therapeutics, Galapagos, GSK, Novartis, Omeros, Otsuka (Visterra) Pharmaceuticals, and Travere Therapeutics, Inc. All other authors have no conflicts of interest to declare.

## Supplementary Material

Supplementary File (PDF) **Supplementary Methods. Supplementary References. Figure S1.** SPARTAN trial patient disposition. **Figure S2.** Mean (SD) UPCR at each study visit. **Figure S3.** UPE per patient at each study visit. **Figure S4.** Mean (SD) eGFR at each study visit. **Figure S5.** Mean (SE) urine biomarkers at each study visit. **Figure S6.** Protein-protein network mapping. **Figure S7.** Mean (SD) office and 24-hour ambulatory BP at baseline and week 6 and change from baseline at week 6. **Table S1.** Mean values and change from baseline in body weight, total body water, and blood glucose at selected study visits. **Table S2.** Mean values at baseline and change from baseline at week 24 in cardiac MRI variables.

## Footnotes

Supplementary file (PDF)

**Supplementary Methods.**

**Supplementary References.**
**Figure S1.** SPARTAN trial patient disposition. **Figure S2.** Mean (SD) UPCR at each study visit. **Figure S3.** UPE per patient at each study visit. **Figure S4.** Mean (SD) eGFR at each study visit. **Figure S5.** Mean (SE) urine biomarkers at each study visit. **Figure S6.** Protein-protein network mapping. **Figure S7.** Mean (SD) office and 24-hour ambulatory BP at baseline and week 6 and change from baseline at week 6. **Table S1.** Mean values and change from baseline in body weight, total body water, and blood glucose at selected study visits. **Table S2.** Mean values at baseline and change from baseline at week 24 in cardiac MRI variables.
