# Supplementary material for: Sparsentan as First-Line Treatment in IgAN From SPARTAN
Source: Kidney Int Rep. 2026 Sep 24;11(12):107105. doi: 10.1016/j.ekir.2026.107105 (PMC13744213; doi:10.1016/j.ekir.2026.107105)
Supplement: Supplementary file 1 [file mmc1.pdf]

Sparsentan as first-line treatment in IgA nephropathy from SPARTAN

Chee Kay Cheung[1,2], William A. Barratt[1], Sulalita Chaki[3], Silpa Chinnakotla[3], Neeraj Dhaun[4,5], Matthew P.M. Graham-Brown[1,6], Siân Griffin[7], Bruce Hendry[8], Alexandra Howson[9], Amal A.A. Jama[1], Wenjun Ju[3], Ishika Khan[1], Radko Komers[10], Matthias Kretzler[11], Alex Mercer[12], Stephanie Moody[13], Viji Nair[14], Nadia Nawaz[1], Kelly Parke[15], Ian S.D. Roberts[16], Matthew Sayer[5], Smeeta Sinha[17,18], Justyna Szklarzewicz[9], Lisa Willcocks[19], Jonathan Barratt[1,2]

[1]Department of Cardiovascular Sciences, University of Leicester, Leicester, United Kingdom; [2]John Walls Renal Unit, University Hospitals of Leicester NHS Trust, Leicester, United Kingdom; [3]Department of Internal Medicine, University of Michigan, Ann Arbor, MI, USA; [4]Institute for Neuroscience and Cardiovascular Research, Edinburgh Kidney, The Queen's Medical Research Institute, University of Edinburgh, Edinburgh, Scotland, United Kingdom; [5]Department of Renal Medicine, Royal Infirmary of Edinburgh, Edinburgh, Scotland, United Kingdom; [6]Division of Cardiovascular Sciences, NIHR Leicester Biomedical Research Centre, Leicester, United Kingdom; [7]Department of Nephrology and Transplantation, University Hospital of Wales, Cardiff, Wales, United Kingdom; [8]Medical Affairs, Travere Therapeutics, Inc., San Diego, CA, USA; [9]Renal Research Unit, University Hospitals of Leicester NHS Trust, Leicester, United Kingdom; [10]Clinical Development, Nephrology, Travere Therapeutics, Inc., San Diego, CA, USA; [11]Medicine/Nephrology and Computational Medicine and Bioinformatics, University of Michigan, Ann Arbor, MI, USA; [12]Clinical Drug Development, JAMCO Pharma Consulting, Stockholm, Sweden; [13]Biostatistics, Travere Therapeutics, Inc., San Diego, CA, USA; [14]Department of Internal Medicine, Division of Nephrology, University of Michigan, Ann Arbor, MI, USA; [15]Department of Cardiovascular Sciences, University Hospitals of Leicester NHS Trust, Leicester, United Kingdom; [16]Department of Cellular Pathology, Oxford University Hospitals NHS Foundation Trust, Oxford, United Kingdom; [17]Division of Cardiovascular Sciences, University of

Manchester, United Kingdom; [18]Department of Renal Medicine, Donal O'Donoghue Renal Research Centre, Northern Care Alliance NHS Foundation Trust, Greater Manchester, United Kingdom; [19]Department of Renal Medicine, Cambridge University Hospitals NHS Trust, Cambridge, United Kingdom

Supplementary Material

Table of Contents

- **Supplementary Methods**
- **Supplementary Figure S1. SPARTAN trial patient disposition**
- **Supplementary Figure S2. Mean (SD) UPCR at each study visit**
- **Supplementary Figure S3. UPE per patient at each study visit**
- **Supplementary Figure S4. Mean (SD) eGFR at each study visit**
- **Supplementary Figure S5. Mean (SE) urine biomarkers at each study visit**
- **Supplementary Figure S6. Protein-protein network mapping**
- **Supplementary Figure S7. Mean (SD) office and 24-hour ambulatory BP at baseline and week 6 and change from baseline at week 6**
- **Supplementary Table S1. Mean values and change from baseline in body weight, total body water, and blood glucose at selected study visits**
- **Supplementary Table S2. Mean values at baseline and change from baseline at week 24 in cardiac MRI variables**
- **Supplementary References**

Supplementary Methods

Patient eligibility criteria

Inclusion criteria

Patients who meet all of the following criteria are eligible for this trial:

1. The patient is willing and able to provide signed informed consent
2. The patient can understand written and spoken English
3. The patient is male or female, aged ≥18 years
4. Diagnosis of IgA nephropathy (IgAN) based on histological analysis of a native kidney biopsy
5. The patient has been diagnosed with biopsy-proven IgAN within the last 6 months (calculated from the date of kidney biopsy, upon which the IgAN-positive diagnosis was made, to the signing of the informed consent form)
6. The patient has a systolic blood pressure (BP) of ≤150 mm Hg and ≥100 mm Hg, and diastolic BP of ≤100 mm Hg and ≥60 mm Hg at screening
7. The patient has a urine total protein value of ≥0.5 g/day at screening
8. The patient has an estimated glomerular filtration rate (eGFR) value of ≥30 mL/min/1.73 m^2 at screening
9. The patient has not previously been treated with angiotensin converting enzyme inhibitors (ACEi) and/or angiotensin receptor blocker (ARB) therapy for IgAN or has not received ACEi and/or ARB therapy within the last 12 months
10. Women of childbearing potential (WOCBP), beginning at menarche, must agree to the use of one highly reliable (ie, can achieve a failure rate of <1% per year) method of contraception from 7 days prior to the first dose of trial medication until 90 days after the last dose of trial medication[a,b]

[a]WOCBP are defined as those who are fertile, following menarche and until becoming postmenopausal unless permanently sterile; permanent sterilization methods include hysterectomy, bilateral salpingectomy, and bilateral oophorectomy. A postmenopausal state is defined as amenorrhea for more than 24 consecutive

months without an alternative medical cause; women on hormone replacement therapy must have a documented plasma follicle-stimulating hormone level ≥40 mIU/mL. All WOCBP must have a negative pregnancy test at visit 1 (serum test) and visit 2 (urine, with positive results confirmed by serum). [b]Highly reliable contraception methods include stable oral, implanted, transdermal, or injected contraceptive hormones associated with inhibition of ovulation, or an intrauterine device in place for at least 3 months. One additional barrier method must also be used during sexual activity, such as a diaphragm or diaphragm with spermicide (preferred), or male partner's use of male condom or male condom with spermicide), from day 1 until 90 days after the last dose of trial medication.

Exclusion criteria

Patients who meet any of the following criteria are excluded from this trial:

1. The patient has IgAN secondary to another condition (eg, systemic lupus erythematosus, liver cirrhosis)
2. Rapidly progressive glomerulonephritis (rapid decline in GFR and crescents on biopsy)
3. History of type 1 diabetes mellitus, uncontrolled type 2 diabetes mellitus, defined by hemoglobin A1c of >8% (>64 mmol/mmol) or non-fasting blood glucose >10 mmol/L (180 mg/dL) at screening
4. The patient has been taking any systemic immunosuppressive medications for >2 weeks within 6 months prior to screening
5. The patient requires any of the prohibited concomitant medications[a]
6. The patient has a documented history of heart failure (New York Heart Association Class II-IV) and/or previous hospitalization for heart failure or unexplained dyspnea, orthopnea, paroxysmal nocturnal dyspnea, ascites, and/or peripheral edema
7. The patient has clinically significant cerebrovascular disease (transient ischemic attack or stroke) and/or coronary artery disease (hospitalization for myocardial infarction or unstable angina, new onset of angina with positive functional tests, coronary angiogram revealing stenosis, or a coronary revascularization procedure) within 6 months prior to screening

8. The patient has jaundice, hepatitis, or known hepatobiliary disease (including asymptomatic cholelithiasis), or alanine aminotransferase or aspartate aminotransferase >2 times the upper limit of the normal range at screening
9. The patient has a history of malignancy other than adequately treated basal cell or squamous cell skin cancer or cervical carcinoma within the past 2 years
10. The patient has a screening hematocrit value of <27% or hemoglobin value of <90 g/L (9 g/dL)
11. The patient has a screening potassium value of >5.5 mmol/L (5.5 mEq/L)
12. The patient has a history of alcohol or illicit drug use disorder (as defined in the Diagnostic and Statistical Manual of Mental Disorders, 5th Edition)
13. The patient has a history of serious side effects or allergic response to any angiotensin or endothelin receptor antagonists, including sparsentan, or has a hypersensitivity to any of the excipients in the investigational medicinal product (IMP)
14. The female patient is pregnant, plans to become pregnant during the course of the trial, or is breastfeeding
15. The patient has participated in a trial of any investigational product within 28 days prior to screening or plans to participate in such a trial during the course of this trial
16. The patient, in the opinion of the Investigator, is unable to adhere to the requirements of the trial, including the ability to swallow the IMP whole
17. The patient, in the opinion of the Investigator, has a medical condition or abnormal clinically significant laboratory screening value not listed above that may interfere with the evaluation of sparsentan safety or activity[b]
18. Patients with a medical condition or abnormal clinically significant laboratory screening value not listed above that may interfere with the evaluation of sparsentan safety or activity will be reviewed before consideration of the patient for enrollment
19. The patient has undergone any organ transplantation, except for corneal transplants

[a]Prohibited medications while taking sparsentan include:

- Inhibitors of the renin-angiotensin-aldosterone system. Examples include (not all inclusive): ACEis, aldosterone blockers, ARBs, spironolactone, eplerenone, aliskiren
- Inhibitors of the endothelin system (bosentan, macitentan, ambrisentan)
- Potassium-sparing diuretics (eg, amiloride, triamterene)
- Selected anti-diabetic drugs (thiazolidinediones) should be avoided completely. Other anti-diabetic drugs (eg, metformin, glyburide) should be used in accordance with their guidelines for use in patients with impaired kidney function
- Digoxin, amiodarone, or any other antiarrhythmic medications that may put the patient at higher risk due to the underlying disease
- Amphetamines, amphetamine derivative agents, and prescribed weight loss medications including orlistat
- St. John's Wort or other hypericum-derived products
- Strong and moderate cytochrome P450 3A (CYP3A) inhibitors and combined strong or moderate CYP3A and P-glycoprotein (P-gp) inhibitors. Coadministration with itraconazole, a combined P-gp and strong CYP3A inhibitor, and cyclosporin, a combined P-gp and moderate CYP3A inhibitor, increase sparsentan area under the curve by 2.7- and 1.7-fold, respectively. Examples of strong CYP3A and P-gp inhibitors include itraconazole, ritonavir, telaprevir, clarithromycin, ketoconazole, and conivaptan. Examples of medications that are moderate CYP3A and P-gp inhibitors include verapamil, erythromycin, diltiazem, and dronedarone. For a detailed list of medications that are strong or moderate CYP3A4 inhibitors, see the P450 Drug Interaction Table at: http://medicine.iupui.edu/clinpharm/ddis/main-table. The study recognizes that, in some cases, concomitant use of these medications may be medically necessary (eg, azole antifungals for severe mycotic infections) and alternatives are either unavailable or inappropriate from a medical and safety perspective. In these cases, the Investigator should contact the Medical Monitor for guidance
- The following medications are prohibited for 7 days prior to trial visits and should be used with caution at other times during the trial. Investigators must review each patient case individually and use clinical judgment
    - Sulfamethoxazole/trimethoprim, cimetidine, pyrimethamine, cetirizine, cobicistat, probenecid, vandetanib, dolutegravir, ranolazine, dronedarone, ritonavir, and telaprevir cannot be used within at least 7 days prior to any visit at which eGFR is assessed
        - Fibrates

[b]Patients with a medical condition or abnormal clinically significant laboratory screening

value not listed above that may interfere with the evaluation of sparsentan safety or activity will be reviewed before consideration of the patient for enrollment.

Study design and assessment schedule

Patients received sparsentan 200 mg/day for the first 2 weeks of treatment before titration to the target dose of 400 mg/day (**Figure 1a**). The 400-mg sparsentan dose was chosen for this trial as an appropriate target dose for treatment of IgAN based on the knowledge of sparsentan pharmacokinetic characteristics and the perceived benefit-risk profile in this progressive disease. At the week 2 visit in SPARTAN, the Investigator evaluates dose tolerance prior to titrating up to the target dose. Dose tolerance is defined as systolic BP >100 mm Hg and diastolic BP >60 mm Hg and no adverse events (AEs) or laboratory findings interfering with the patient's continuation on sparsentan. To maintain BP as close as possible to the target of 125/75 mm Hg, the Investigator is encouraged to either treat patients with additional antihypertensive agents, excluding ACEis, aldosterone blockers, aliskiren, or ARBs or withdraw antihypertensive agents other than sparsentan. In the case of intolerable hypotension, withdrawal of additional antihypertensive agents should be done before considering withdrawal or dose reduction of sparsentan. Patients followed a schedule of routine trial assessments from screening through week 24 (**Figure 1b**).

Magnetic resonance imaging (MRI) analysis methodology

Image analysis was conducted blinded offline to all participant details by an experienced single observer using Circle CVI (Circle Cardiovascular Imaging, cvi42 V6.1.2 Calgary, Alberta, Canada) and Java image manipulation (JIM) v 8.0 (Xinapse Systems). All scans were performed using a 3-Tesla (Magnetom Vida or Skyra, Siemens Healthineers, Erlangen, Germany) with electrocardiographic gating, an anterior 18-channel phased-array cardiac receiver coil, and a posterior 32-channel spine array.

Breath-held, steady-state free-procession cine imaging of the 4 chamber, 2 chamber, 3 chamber, and short axis stack to cover the left and right ventricles were acquired for functional imaging. Scans were performed with either retrospective or prospective gating, depending on electrocardiogram rhythm at time of scan. Typical sequence parameters: slice thickness 8 mm, distance factor 25%, repetition time 48 ms, echo time 1.5 ms, matrix 166 x 256, field of view (FOV) 340 mm, FOV phase 81.3%, and flip angle 54°.

Native T1 maps were acquired within the heart in the base, mid, and apical short axis view. In the right and left kidney, 3 axial images were obtained in the superior, mid, and inferior position. All images were acquired breath held with 3(3)3(3)5 sampling pattern with typical sequence parameters: slice thickness 8.0mm, FOV 360 mm, FOV phase 85.2%, flip angle 35°, and minimum TI 180ms, as previously described.[1,2]

Left ventricular mass and left and right ventricular function and volumes were calculated using automated rounded epicardial and endocardial borders (excluding papillary muscles) at both the end-systolic and end-diastolic phases, with manual checking and correction of the basal and apical slices. Left ventricular systolic and diastolic strain rates were calculated by cardiovascular magnetic resonance feature tracking using the cvi42 Tissue Tracking module to derive longitudinal and circumferential peak early diastolic strain rates, global longitudinal strain, and global circumferential strain.

Cardiac short axis T1 maps were analyzed using Cvi42 manual contours of the epicardial and endocardial borders; a 10% offset was added to ensure only the myocardium was

included. Manual contouring of the cortex of the right and left kidney was obtained in each slice, ensuring to exclude artefact, as previously described.[2]

Aortic distensibility was measured using a breath-held steady-state free precession cine performed perpendicular to the aorta at the level of the pulmonary artery. BP was taken at the same time to calculate the pulse pressure. In JIM, every sixth image was manually contoured with propagation of the other phases, with manual review and adjustment of automated contours as previously described.[3] The below calculation was then applied to work out the aortic distensibility:

$$\frac{(\text{maximum aortic area} - \text{minimum aortic area})}{(\text{minimum aortic area X pulse pressure})}$$

Enzyme-Linked immunosorbent assay (ELISA) measurements

Assays (BD OptEIA™ ELISA, BD Biosciences, San Diego, CA, USA; DuoSet ELISA, R&D Systems, Bio-Techne, Minneapolis, MN, USA; Human BAFF [TNFSF13B] ELISA Kit, Human CXCL16 [Chemokine C-X-C-Motif Ligand 16] ELISA Kit, and Human Growth/differentiation factor 15 [GDF15] ELISA Kit, Assay Genie, Dublin, Ireland; Creatinine Urinary Detection Kit, Invitrogen, Thermo Fisher Scientific, Carlsbad, CA, USA) were performed according to the manufacturers' instructions. Samples were analyzed in duplicate, and measurements with a coefficient of variation (CV) >10% were repeated. For samples with low analyte concentrations, near the lower limit of quantification (LLOQ), higher CVs were accepted where both replicate optical density values fell below the second lowest standard. This was because CVs are disproportionally increased at low signal intensity with minimal changes to interpolated concentrations. Non-baseline values below the LLOQ were imputed as LLOQ/2. GraphPad Prism, version 10.2.3, was used to interpolate analyte concentrations from the assay-specific standard curve. All analytes were normalized to urinary creatinine. Changes from baseline for each analyte were analyzed. Patients with undetectable baselines were excluded from the analysis, as a quantifiable baseline value is required to calculate a meaningful change score.

Concentrations of alpha-2-macroglobulin (Human alpha 2-Macroglobulin DuoSet ELISA [DY1938], R&D Systems, Bio-Techne, Minneapolis, MN, USA), clusterin (Human Clusterin ELISA Kit [ab174447], Abcam, Waltham, MA, USA), and plasminogen (Human Plasminogen ELISA Kit [PLG, ab108893], Abcam, Waltham, MA, USA) were measured in urine samples according to standard or modified standard protocols at dilutions of 1:10, 1:32, and 1:32, respectively. Quality control samples containing high, medium, and low concentrations of the respective analytes were included in each ELISA plate to monitor batch effects. The inter-assay CVs of the quality controls for the above-mentioned biomarkers were 6.1%, 2.03%, and 3.2%, respectively.

For samples below the limit of quantification (BLQ), concentrations calculated from a detectable optical density were retained when the CV between duplicate measurements was <20%. For BLQ samples without a calculated concentration, values were imputed as 50% of the lowest reported BLQ concentration within the same assay. The same imputed value was assigned to all BLQ samples without detectable concentrations within that assay. Biomarker concentrations, including imputed values, were normalized to urinary creatinine to correct for hydration status and reported as pg/mg or, where appropriate to facilitate interpretation, converted to ng/mg. Log_2 transformation was applied for downstream analyses to improve the normality of the data distribution.

Protein-protein network mapping analysis

Protein-protein interaction network analysis, identification of gene ontology annotations, and functional analyses were performed using STRINGdb v11 and the Kyoto Encyclopedia of Genes and Genomes pathways as the source for annotations.[4]

Supplementary Figures

Supplementary Figure S1. SPARTAN trial patient disposition

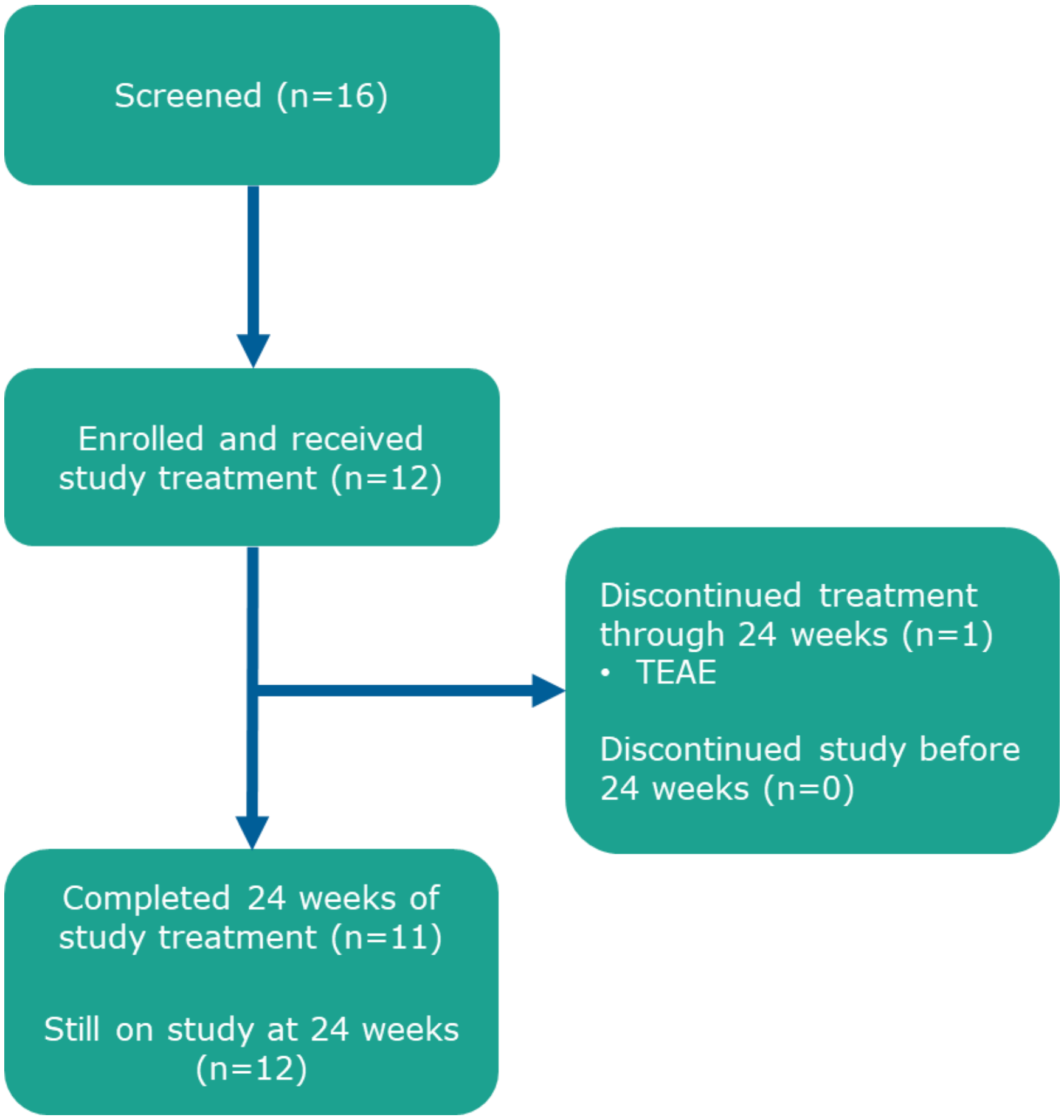

Supplementary Figure S2. Mean (SD) UPCR at each study visit

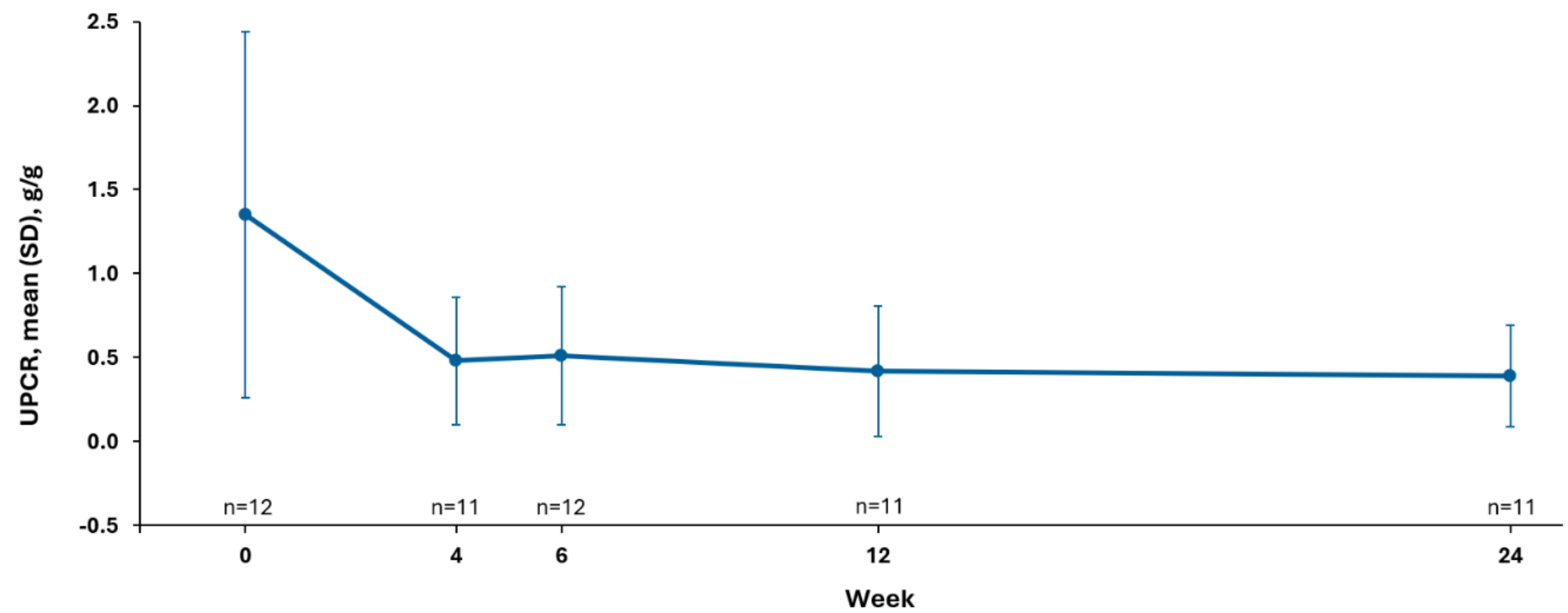

SD, standard deviation; UPCR, urine protein-to-creatinine ratio.

Supplementary Figure S3. UPE per patient at each study visit

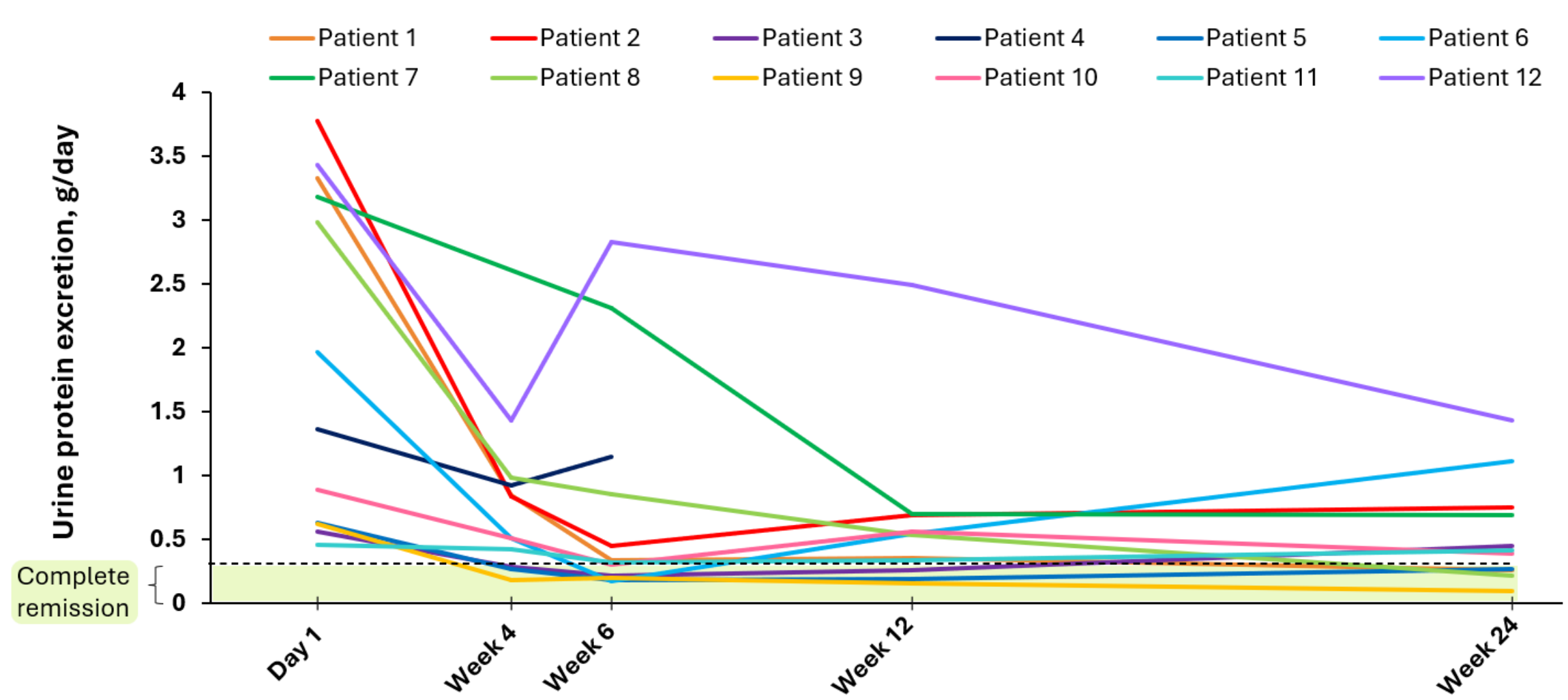

UPE, urine protein excretion.

Supplementary Figure S4. Mean eGFR (SD) at each study visit[a]

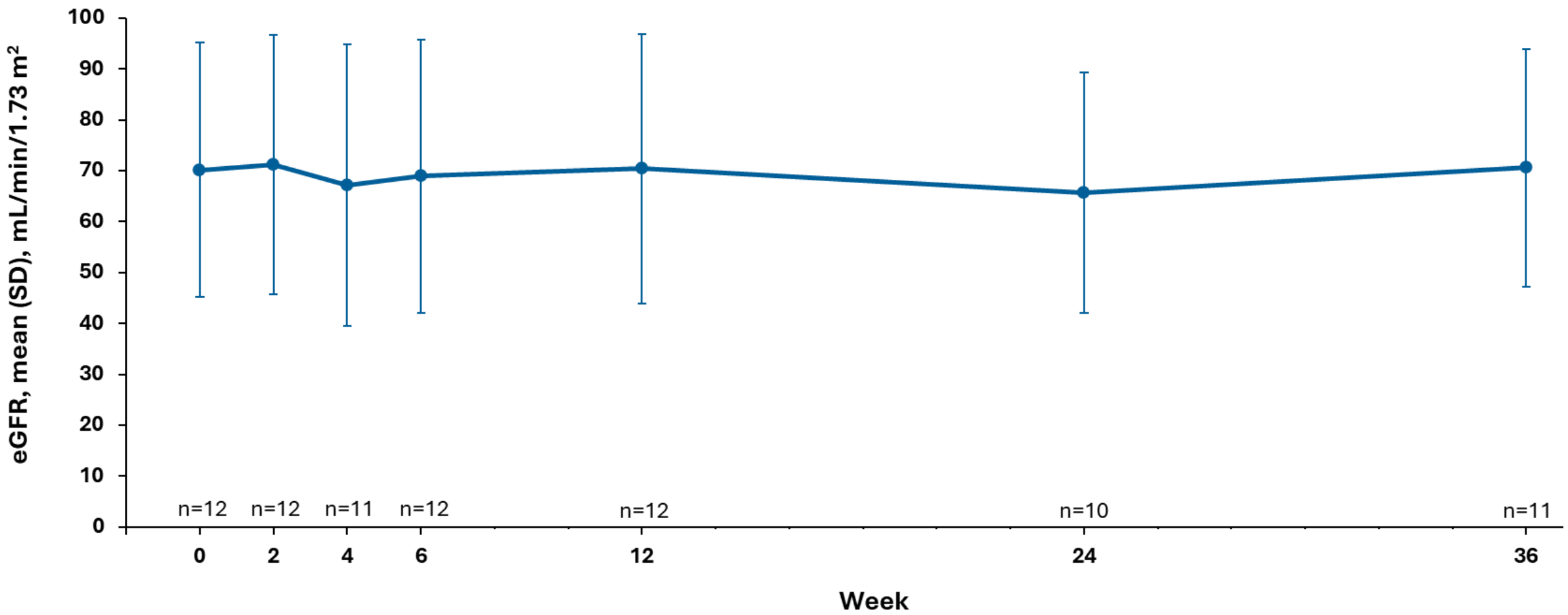

[a]Data from baseline to week 36 are presented to show the stability of eGFR over the full period of the interim analysis.

eGFR, estimated glomerular filtration rate; SD, standard deviation.

Supplementary Figure S5. Mean (SE) urine biomarkers at each study visit. Mean absolute values in (a) BAFF, (b) sC5b9, (c) sCD163, (d) α2M, (e) CHI3L1, (f) clusterin, (g) GDF15, (h) plasminogen, (i) CXCL10, (j) CXCL16, (k) IL6, and (l) MCP-1 at each study visit Blue, immune cell modulation biomarkers; teal, inflammatory and profibrotic biomarkers; green, chemokine and cytokine biomarkers.

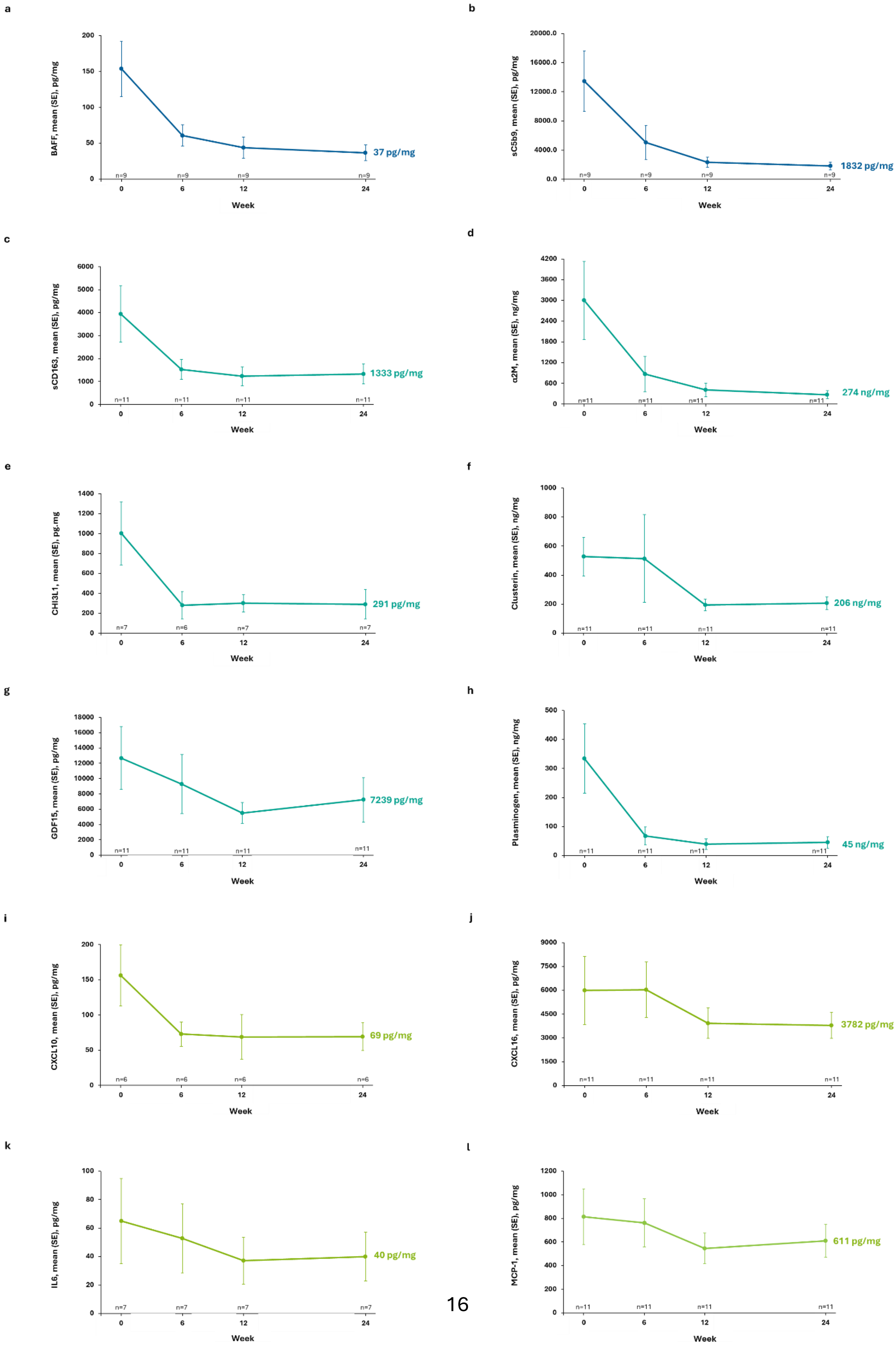

a
BAFF, mean (SE), pg/mg
37 pg/mg
n=9
Week
b
sC5b9, mean (SE), pg/mg
1832 pg/mg
n=9
Week
c
sCD163, mean (SE), pg/mg
1333 pg/mg
n=11
Week
d
α2M, mean (SE), ng/mg
274 ng/mg
n=11
Week
e
CHI3L1, mean (SE), pg.mg
291 pg/mg
n=7
n=6
Week
f
Clusterin, mean (SE), ng/mg
206 ng/mg
n=11
Week
g
GDF15, mean (SE), pg/mg
7239 pg/mg
n=11
Week
h
Plasminogen, mean (SE), ng/mg
45 ng/mg
n=11
Week
i
CXCL10, mean (SE), pg/mg
69 pg/mg
n=6
Week
j
CXCL16, mean (SE), pg/mg
3782 pg/mg
n=11
Week
k
IL6, mean (SE), pg/mg
40 pg/mg
n=7
Week
l
MCP-1, mean (SE), pg/mg
611 pg/mg
n=11
Week

α2M, alpha-2-macroglobulin; BAFF, B-cell activating factor; CHI3L1, chitinase-3-like protein 1; CXCL10, c-x-c motif chemokine ligand 10; CXCL16, c-x-c motif chemokine ligand 16; GDF15, growth/differentiation factor 15; IL6, interleukin-6; MCP-1, monocyte chemoattractant protein-1; sC5b9, soluble C5b9; sCD163, soluble CD163; SE, standard error.

Supplementary Figure S6. Protein-protein network mapping

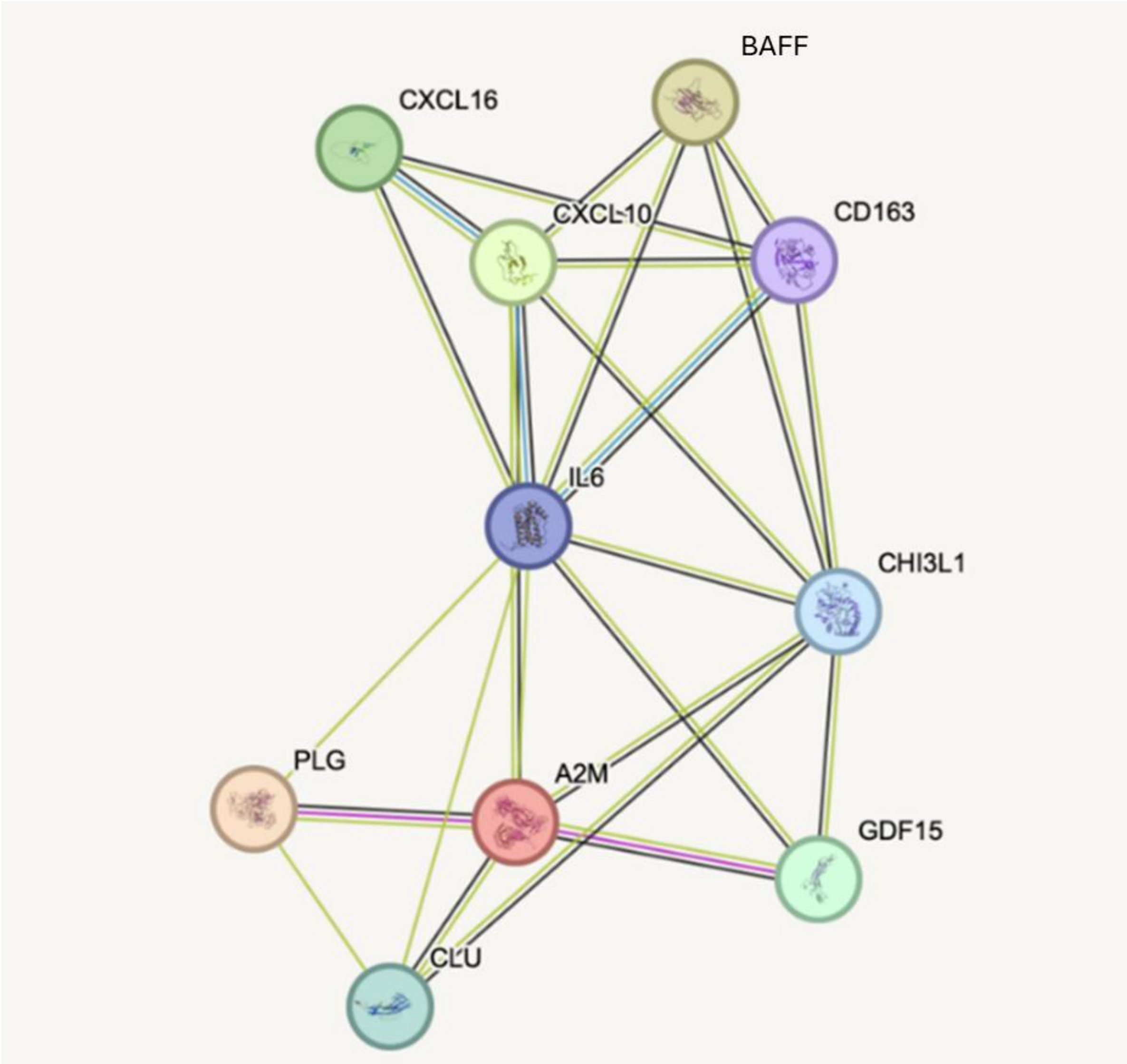

A2M, alpha-2-macroglobulin; BAFF, B-cell activating factor; CHI3L1, chitinase-3-like protein 1; CLU, clusterin; CXCL10, c-x-c motif chemokine ligand 10; CXCL16, c-x-c motif chemokine ligand 16; GDF15, growth/differentiation factor 15; IL6, interleukin-6; PLG, plasminogen.

Supplementary Figure S7. Mean (SD) office and 24-hour ambulatory BP at baseline and week 6 and change from baseline at week 6

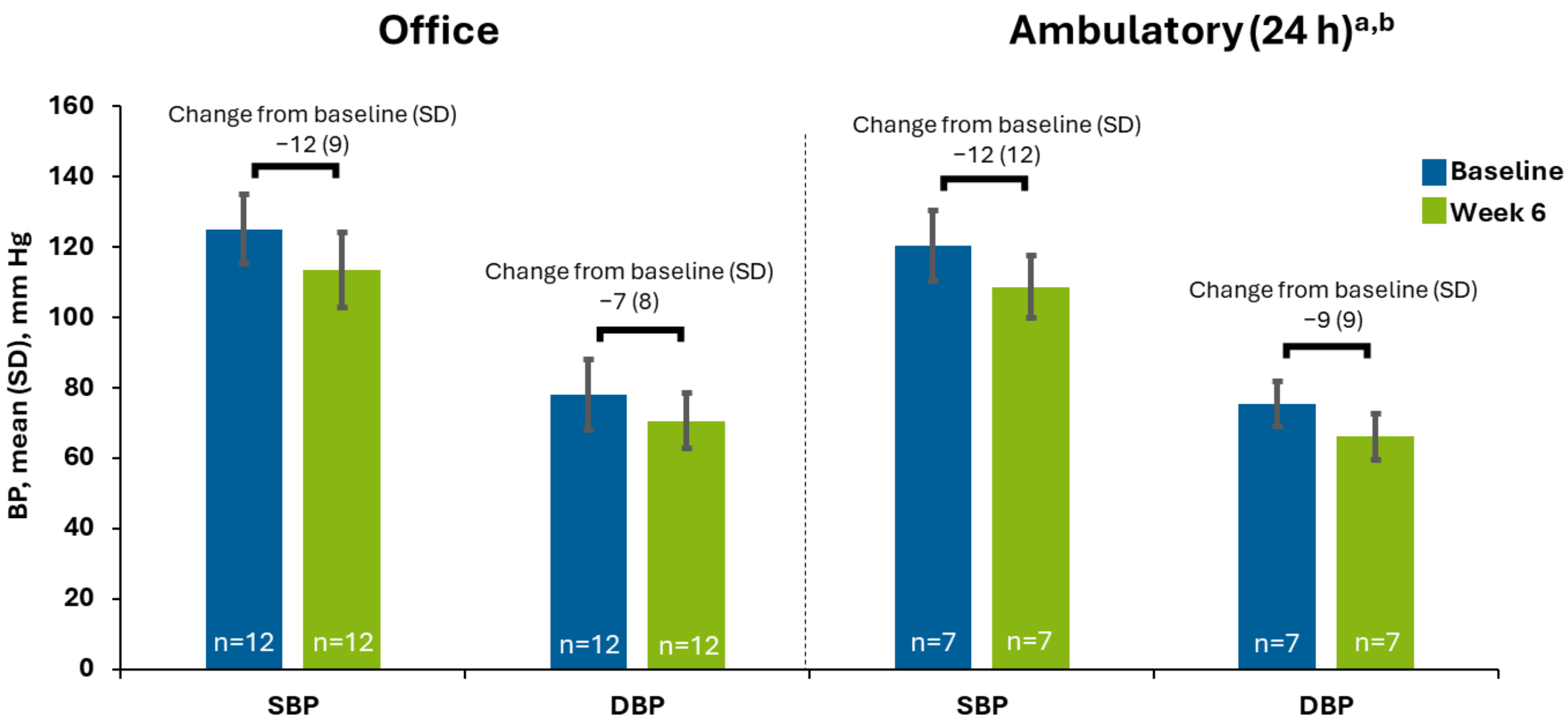

[a]Patients who had ambulatory BP data available at baseline and week 6. [b]Measured over 24 hours.

BP, blood pressure; DBP, diastolic blood pressure; SBP, systolic blood pressure; SD, standard deviation.

Supplementary Tables

Supplementary Table S1. Mean values and change from baseline in body weight, total body water, and blood glucose at selected study visits

	Body weight, kg		Total body water, L		Blood glucose, mmol/L	
	Mean values (SD)	Mean change from baseline (SD)	Mean values at (SD)	Mean change from baseline (SD)	Mean values at (SD)	Mean change from baseline (SD)
Baseline (n=12)	83.1 (24.7)	Reference	47.1 (7.4)	Reference	4.7 (0.6)	Reference
Week 2 (n=12)	82.8 (24.6)	−0.3 (0.7)	–[a]	–[a]	4.8 (0.5)	0.1 (0.7)
Week 4 (n=11)	82.4 (25.4)	−0.2 (1.4)	–[a]	–[a]	–[b]	–[b]
Week 6 (n=12)	83.4 (24.2)	0.3 (1.4)	45.2 (10.6)	-2.0 (7.2)	4.8 (0.8)	0.2 (0.7)
Week 12 (n=11)	85.9 (22.9)	0.1 (2.7)	–[a]	–[a]	4.7 (0.3)	−0.2 (0.3)
Week 24 (n=11)	84.8 (22.9)	−1.0 (3.3)	45.3 (8.5)	−2.4 (7.4)	4.8 (0.3)	0.2 (0.6)

[a]Total body water was not assessed at weeks 2, 4, and 12. [b]Blood glucose was not assessed at week 4.

SD, standard deviation

Supplementary Table S2. Mean and change from baseline at week 24 in cardiac MRI variables

Cardiac variable, mean (SD)	Values at baseline (n=7)	Change from baseline at week 24 (n=7)
Left ventricular end-diastolic volume, mL	167.1 (38.4)	5.6 (13.6)
Left ventricular end-diastolic volume index, mL/m^2	86.6 (17.2)	1.9 (5.9)
Left ventricular end-systolic volume, mL	68.6 (11.2)	3.4 (17.7)
Left ventricular end-systolic volume index, mL/m^2	35.7 (5.8)	0.6 (8.2)
Left ventricular stroke volume, mL/beat	98.5 (29.7)	2.1 (5.6)
Left ventricular cavity obliteration, ms	6.7 (1.7)	−0.5 (1.0)
Left ventricular ejection fraction, %	58.3 (4.9)	0.3 (6.6)
Left ventricular mass, g	105.4 (30.1)	−5.5 (8.5)
Left ventricular mass index, g/m^2	53.8 (9.9)	−3.1 (3.5)
Right ventricular end-diastolic volume, mL	184.9 (54.8)	2.5 (18.0)
Right ventricular end-diastolic volume index, mL/m^2	95.3 (24.2)	0.4 (8.8)
Right ventricular end-systolic volume, mL	88.9 (32.8)	3.6 (21.2)
Right ventricular end-systolic volume index, mL/m^2	96.0 (26.0)	−1.1 (4.5)
Right ventricular cavity obliteration, ms	6.5 (1.5)	−0.7 (1.0)
Right ventricular ejection fraction, %	52.4 (6.1)	−1.2 (6.3)
Global circumferential strain, %	−18.0 (2.0)	−0.5 (1.3)
Global longitudinal strain, %[a]	−17.1 (1.7)	−1.2 (1.9)
Peak early diastolic circumferential strain rate, s^{-1}	0.7 (0.1)	−0.03 (0.1)
Peak late diastolic circumferential strain rate, s^{-1}	0.4 (0.1)	−0.02 (0.2)
Native T1, ms[a]	1291.1 (54.5)	12.9 (35.1)
Native T2, ms[a]	41.4 (4.3)	2.3 (3.8)

[a]n=8.

MRI, magnetic resonance imaging; SD, standard deviation.

Supplementary References

S1. Graham-Brown MP, Rutherford E, Levelt E, et al. Native T1 mapping: inter-study, inter-observer and inter-center reproducibility in hemodialysis patients. *J Cardiovasc Magn Reson*. 2017;19(1):21. doi:10.1186/s12968-017-0337-7

S2. Graham-Brown MP, Singh A, Wormleighton J, et al. Association between native T1 mapping of the kidney and renal fibrosis in patients with IgA nephropathy. *BMC Nephrol*. 2019;20(1):256. doi:10.1186/s12882-019-1447-2

S3. Graham-Brown MPM, Adenwalla SF, Lai FY, et al. The reproducibility of cardiac magnetic resonance imaging measures of aortic stiffness and their relationship to cardiac structure in prevalent haemodialysis patients. *Clin Kidney J*. 2018;11(6):864–873. doi:10.1093/ckj/sfy042

S4. Wimbury D, Muto M, Bhachu JS, et al. Targeted-release budesonide modifies key pathogenic biomarkers in immunoglobulin A nephropathy: insights from the NEFIGAN trial. *Kidney Int*. 2024;105(2):381–388. doi:10.1016/j.kint.2023.11.003
